# Characterization of cell-type specific circular RNAs associated with colorectal cancer metastasis

**DOI:** 10.1093/narcan/zcad021

**Published:** 2023-05-19

**Authors:** Sidi Zhao, Amy Ly, Jacqueline L Mudd, Emily B Rozycki, Jace Webster, Emily Coonrod, Ghofran Othoum, Jingqin Luo, Ha X Dang, Ryan C Fields, Christopher A Maher

**Affiliations:** Department of Internal Medicine, Washington University School of Medicine, St Louis, MO 63108, USA; Department of Internal Medicine, Washington University School of Medicine, St Louis, MO 63108, USA; Department of Surgery, Washington University School of Medicine, St Louis, MO 63108, USA; Department of Internal Medicine, Washington University School of Medicine, St Louis, MO 63108, USA; Department of Internal Medicine, Washington University School of Medicine, St Louis, MO 63108, USA; Department of Internal Medicine, Washington University School of Medicine, St Louis, MO 63108, USA; Department of Internal Medicine, Washington University School of Medicine, St Louis, MO 63108, USA; Alvin J. Siteman Cancer Center, Washington University School of Medicine, St Louis, MO 63108, USA; Division of Public Health Sciences, Department of Surgery, Washington University School of Medicine, St Louis, MO 63108, USA; Department of Internal Medicine, Washington University School of Medicine, St Louis, MO 63108, USA; Alvin J. Siteman Cancer Center, Washington University School of Medicine, St Louis, MO 63108, USA; Department of Surgery, Washington University School of Medicine, St Louis, MO 63108, USA; Department of Internal Medicine, Washington University School of Medicine, St Louis, MO 63108, USA; Alvin J. Siteman Cancer Center, Washington University School of Medicine, St Louis, MO 63108, USA; Department of Biomedical Engineering, Washington University School of Medicine, St Louis, MO 63108, USA

## Abstract

Colorectal cancer (CRC) is the most common gastrointestinal malignancy and a leading cause of cancer deaths in the United States. More than half of CRC patients develop metastatic disease (mCRC) with an average 5-year survival rate of 13%. Circular RNAs (circRNAs) have recently emerged as important tumorigenesis regulators; however, their role in mCRC progression remains poorly characterized. Further, little is known about their cell-type specificity to elucidate their functions in the tumor microenvironment (TME). To address this, we performed total RNA sequencing (RNA-seq) on 30 matched normal, primary and metastatic samples from 14 mCRC patients. Additionally, five CRC cell lines were sequenced to construct a circRNA catalog in CRC. We detected 47 869 circRNAs, with 51% previously unannotated in CRC and 14% novel candidates when compared to existing circRNA databases. We identified 362 circRNAs differentially expressed in primary and/or metastatic tissues, termed circular RNAs associated with metastasis (CRAMS). We performed cell-type deconvolution using published single-cell RNA-seq datasets and applied a non-negative least squares statistical model to estimate cell-type specific circRNA expression. This predicted 667 circRNAs as exclusively expressed in a single cell type. Collectively, this serves as a valuable resource, TMECircDB (accessible at https://www.maherlab.com/tmecircdb-overview), for functional characterization of circRNAs in mCRC, specifically in the TME.

## Graphical Abstract

**Graphical Abstract:**
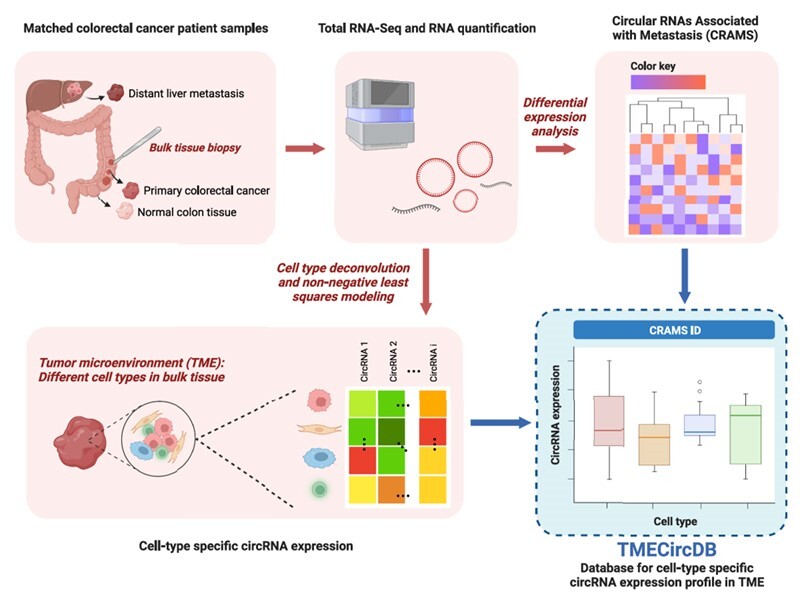

## INTRODUCTION

Tumor growth in the colon and rectum, classified as colorectal cancer (CRC), currently ranks third in terms of incidence and second in terms of mortality globally (1,2). In the United States, CRC is the most common gastrointestinal malignancy, constituting almost 10% of all cancer cases and deaths. Approximately 60% of patients with CRC develop metastatic disease (mCRC), most commonly in the liver (2–4). Although early-stage CRC is curable with surgery and adjuvant therapy, mCRC is usually lethal, with a 5-year survival rate of 13% (5).

To date, CRC research has primarily focused on the dysregulation of protein-coding genes to identify oncogenes and tumor suppressors as potential diagnostic and therapeutic targets (6). One emerging class of noncoding RNAs, circular RNAs (circRNAs), has been shown to be functionally implicated in CRC tumorigenesis. CircRNAs are single-stranded circular molecules averaging 500 nucleotides in length, with a covalent 3′–5′ bond formed in the process of backsplicing, where a downstream splice donor on a pre-mRNA pairs with an unspliced upstream splice acceptor (7–9). Though intron retention can happen in a small percentage of circRNAs, exonic circRNAs make up the majority of annotated circRNAs (10,11). The lack of an open 3′ end makes circRNAs particularly stable compared to their linear counterparts because they are resistant to exonucleolytic decay by the cellular exosome ribonuclease complex (11). Moreover, the higher stability and longer half-life of circRNAs relative to mRNAs allow them to accumulate in exosomes and blood, thereby making them promising potential biomarkers for noninvasive detection of cancer metastasis (12–16). Therefore, characterizing circRNAs, elucidating their function and assessing their clinical applicability could significantly impact cancer diagnosis and treatment.

CircRNAs have been found to affect a diverse range of molecular events, with their most heavily theorized function being microRNA (miRNA) sponges that counteract their target miRNA’s inhibitive effects (11). However, most reported miRNA sponges were derived from bulk RNA sequencing (RNA-seq) data, without accounting for the cell-type specificity of the circRNA and miRNA involved, i.e. the spatial origins of a circRNA in the tumor microenvironment (TME). The TME is described as the ‘soil’ for metastatic tumor cell growth, as the intricate crosstalk between cancer cells and their surrounding structure contributes greatly to tumor initiation, development and therapeutic response (17). CiRS-7 is one of the most well-documented miRNA sponges, but, strikingly, Kristensen *et al.* discovered that it may be able to function through alternative mechanisms depending on its specific location within the TME. They reported that ciRS-7 was completely absent in CRC cancer cells, but highly expressed in stromal cells. The putative ‘sponging’ target miR-7, on the other hand, was only found to be expressed in cancer cells, rendering the miRNA sponge mechanism implausible (18). As this discovery poses significant challenge to the prevailing miRNA sponge hypothesis, it is evident that the spatial origin of circRNAs should be determined first before any functional investigation.

Many recent studies have shown that many circRNAs are implicated in crucial hallmarks of CRC tumorigenesis, such as sustaining proliferative signaling, avoiding immune destruction, activating invasion and metastasis, and evading cell death and senescence (4,14,19–58). While transcriptome sequencing has provided an unbiased method for discovering circRNAs, existing large-scale sequencing projects such as The Cancer Genome Atlas Network predominantly consist of primary tumors, thereby lacking matched metastatic samples (59). This represents a critical barrier to discovering circRNAs altered throughout the progression of primary to metastatic disease.

Furthermore, multiple obstacles have yet to be overcome in the study of cell-type specific expression of circRNAs. Most known single-cell RNA-seq (scRNA-seq) protocols require poly(A) selection, without which the sequencing data tend to be very noisy (60). As circRNAs lack open ends, poly(A) selection renders capturing circRNAs difficult. Existing digital flow cytometry tools that enumerate cell-type composition or infer cell-type specific gene expression such as CIBERSORT are typically limited to comparing bulk gene expression profiles with gene signatures established from the linear transcriptome, without representing circRNAs. Hence, the scarcity of scRNA-seq data that include circRNA cell-type markers has contributed to a lack of ground truths of cell-type specific circRNA expression (61,62).

To address this, we performed RNA-seq of matched normal, primary and distant metastatic tissues from CRC patients and conducted a meta-analysis to discover differentially expressed (DE) circRNAs in metastatic tumors compared to primary tumors, termed circular RNAs associated with metastasis (CRAMS). Further, to overcome the limitations of determining cell-type specific expression of circRNAs in the TME, we performed cell-type deconvolution followed by a non-negative least squares (NNLS) model to estimate cell-type specific expression of circRNAs (62,63). From the integrative analysis results, we highlighted circRNAs that showed significant dysregulation and/or cell-type specificity in mCRC progression. The cell-type specific expression data of identified circRNAs are available at the TMECircDB resource: https://www.maherlab.com/tmecircdb-overview.

## MATERIALS AND METHODS

### Patient samples and total RNA-seq

To evaluate circRNAs in CRC metastatic progression, we collected specimens from mCRC patients and performed RNA-seq. Patients (*N* = 14) were enrolled at Washington University School of Medicine in St Louis (WUSTL) and informed consent was obtained under an Institutional Review Board (IRB)-approved protocol. Adjacent normal colon [normal (N); *n* = 8], primary CRC [primary (P); *n* = 6], liver metastasis [metastasis (M); *n* = 13] and normal liver (NL; *n* = 3) tissues were resected from 14 stage IV mCRC patients and fresh frozen prior to RNA extraction. Input RNA quantity was 1 μg except for samples 15 (0.5 μg) and 27 (0.24 μg) due to low volume (Supplementary Table S1). RNA-seq libraries were constructed using the Illumina TruSeq Stranded Total RNA Library Prep kit with IDT xGen Dual Index UMI Adapters. Paired-end sequencing was performed on Illumina HiSeq X.

### Cell culture and cDNA-capture RNA-seq

To evaluate circRNA expression in cell lines, we performed RNA-seq of five cell lines commonly used in CRC research. HCT116 and HT29 cells were grown in McCoy’s medium (Invitrogen, Carlsbad, CA) supplemented with 10% fetal bovine serum and 1% penicillin/streptomycin. SW480 and SW620 cells were grown in DMEM (Invitrogen) supplemented with 10% fetal bovine serum (Sigma, St Louis, MO) and 1% penicillin/streptomycin. LoVo cells were grown in DMEM/F12 (Invitrogen) supplemented with 10% fetal bovine serum and 1% penicillin/streptomycin. Total RNA inputs of 1 μg were used to generate libraries using the New England Biolabs NEB Ultra II Directional RNA Library Prep for Illumina with rRNA Depletion kits. To construct the cell line reference database, 200 ng aliquots of each library were pooled and used as input for cDNA capture. The IDT xGen Exome Research Panel v2 including 415 115 exon probes was used for exome capture and paired-end sequencing was performed on Illumina HiSeq X.

For each cell line sample, uniquely mapped reads and spliced reads were compared with exon probes from IDT xGen Exome Research Panel v2 using BEDtools v2.29.0 overlap to identify on- and off-target reads that were subsequently used to calculate cDNA-capture sequencing on-target rates (64).

### RNA-seq read alignment and circRNA quantification

RNA-seq reads were aligned to the human genome reference assembly version GRCh38. Gene annotations were combined from Gencode v23, RefSeq downloaded from the UCSC Genome Browser and the Broad long noncoding RNA (lncRNA) catalog as described in Silva-Fisher *et al.* (65). Redundant transcripts were removed and overlapping transcripts were assigned to the same gene.

As backspliced reads do not map to the linear transcriptome, chimeric alignments were extracted as a BED file from alignment results via STAR v2.7.3a (66). The chimeric reads were then used to quantify circRNAs using CIRCexplorer2 v2.3.8 (67). For downstream analysis purposes, linear genes were also quantified using featureCounts v1.6.0 (68). Alternative haplotypes were filtered out.

### Existing circRNA database collection

A total of 740 513 nonredundant circRNAs were downloaded from circAtlas v2.0, which encompasses annotations (with necessary liftover from previous genome assemblies) from circbank, circBase, CIRCpedia v2, CircRiC, circRNADb, MiOncoCirc, deepBase v2.0, TCSD and CSCD (69–77). Additionally, 18 447 circRNAs present in the CRC patient cohort of MiOncoCirc were used as the basis of comparison with circRNAs detected in our patient samples (74). When comparing backsplice junctions, 1-bp adjustments were made according to how each database annotates junction boundaries.

### DE analysis

To identify circRNAs associated with CRC tumor and metastatic progression, DE analysis was carried out comparing normal, primary and metastatic tissues via edgeR v3.30.3, using raw counts of linear genes and circRNAs (78). All linear and circular transcripts were combined and prefiltered [≥5 transcript per million (TPM) in 25% of all samples for linear genes; ≥1 backspliced read in 15% of all samples for circRNAs]. All transcripts were compared between primary versus normal (PvN), metastasis versus primary (MvP) and metastasis versus normal samples (MvN). A circRNA with *P* < 0.05, |log fold change (FC)| > 0 and FDR adjusted *P* ≤ 0.15 was considered differentially expressed.

As means of orthogonal validation, the CIRI_DE_replicate function of CIRIquant v1.1.2 (with prerequisites: BWA v0.7.17, HISAT2 v2.2.0 and StringTie v2.1.1) and the R package CircTest v0.1.1 were also run on the same cohort of detected circRNAs (79,80). CircRNAs characterized by CIRIquant to be differentially expressed (*P* < 0.05) in the same comparison group and in the same direction (up- or downregulated) and/or circRNAs found by CircTest to be differentially expressed (*P* < 0.05) in the same comparison group were considered validated in parallel.

### Cell-type deconvolution

**Figure 1.**
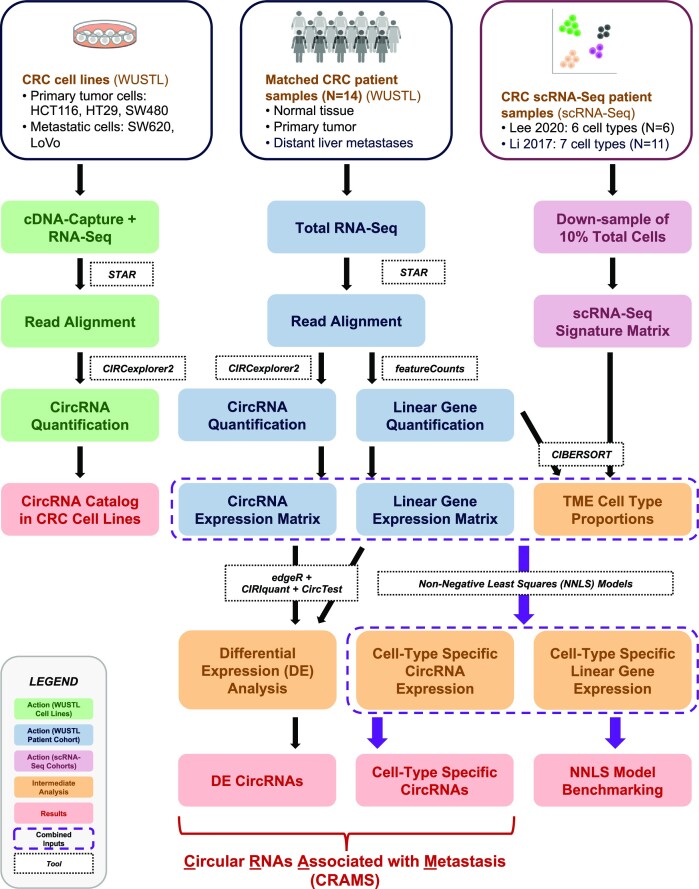
Overview of experimental design. Bulk mCRC patient samples are referred to as the WUSTL patient cohort; CRC scRNA-seq samples from Lee *et al.* and Li *et al.* are referred to as the Lee 2020 scRNA-seq cohort and the Li 2017 scRNA-seq cohort, respectively. Five CRC cell line samples (termed WUSTL cell lines) were enriched for exonic circRNAs through cDNA capture before RNA-seq, followed by computational analysis to quantify circRNAs. CircRNAs catalogued in cell lines will be used for future validation of highlight candidates discovered in patient samples. Thirty matched CRC patient samples from 14 patients underwent total RNA-seq, whereby both circRNAs and linear genes were quantified and normalized to construct expression matrices. DE analysis was performed to identify circRNAs with altered expression in mCRC. Six patient samples from the Lee 2020 scRNA-seq cohort and 11 patient samples from the Li 2017 scRNA-seq cohort were used to build two separate signature matrices to train CIBERSORT, thereby estimating cell-type proportions in TME and cell-type specific linear gene expression, respectively. The circRNA expression matrix and the TME cell-type proportions were used to estimate cell-type specific circRNA expression via NNLS statistical models. Likewise, cell-type specific linear gene expression was estimated using the linear gene expression matrix and TME proportions. The NNLS model estimations were benchmarked by comparing reconstructed ‘bulk’ expression of circRNAs and linear genes with the ground truth values. Collectively, DE and cell-type specific circRNAs were identified as CRAMS.

To estimate the cell-type composition of the WUSTL patient cohort, we built scRNA-seq-based signature matrices for deconvolution with CIBERSORT’s ‘Create Signature Matrix’ module using scRNA-seq datasets of six Belgian patients downloaded from Lee *et al.* (81) (GEO accession code GSE144735) and scRNA-seq datasets from 11 Singaporean patient samples downloaded from Li *et al.* (82) (GEO accession code GSE81861) (Figure 1, right column). To optimize runtime, a random downsample of 10% of all cells while keeping ground truth cell-type proportions was used (Supplementary Table S4). The Lee 2020 scRNA-seq cohort encompassed six major cell types in the CRC TME, namely T cells, B cells, epithelial cells, mast cells, myeloid cells and stromal cells (Supplementary Figure S2A and Supplementary Table S4); the Li 2017 scRNA-seq cohort included seven cell types, namely T cells, B cells, epithelial cells, mast cells, macrophages, endothelial cells and fibroblasts (Supplementary Figure S2B and Supplementary Table S4). Using both datasets in parallel, the bulk linear gene expression (in TPM) of the WUSTL patient cohort was processed through CIBERSORT’s ‘Impute Cell Fractions’ module to estimate cell-type composition (Figure 5A, steps 3–5, Supplementary Figure S3A, and Supplementary Table S5) (62,81,82).

### Cell-type expression specificity analysis

Cell-type proportion estimates from CIBERSORT were used to build an NNLS statistical model to estimate the cell-type specific expression value of circRNAs based on their bulk expression (in normalized backspliced reads) values (Figure 5A, step 6). The Lawson–Hanson algorithm for the NNLS model using R package nnls v1.4 was applied for each sample to produce the estimated coefficients of cell-type specific expression for each circRNA as well as each linear gene (Figure 5A and Supplementary Figure S3A). In short, each transcript’s bulk expression was modeled as a constrained linear least squares regression of the cell-type proportions where all estimated coefficients in the regression must be non-negative (Supplementary Figure S3A) (63). Two models were constructed for both scRNA-seq references, termed the Lee 2020 model and the Li 2017 model. For benchmarking purposes, both models were also run to estimate cell-type specific linear gene expression values.

### Computational model benchmarking

To assess the performance of the NNLS models, we reconstructed ‘bulk’ expression from each model’s estimated cell-type specific expression of both linear genes and circRNAs, which was then compared with the ground truth to evaluate how accurately the model recaptures the real expression values (Figure 5B–E, Supplementary Figure S3B and C, and Supplementary Tables S7 and S8).

For linear genes, modeled ‘bulk’ expression (matrix *L*) of every gene in every sample was calculated through matrix multiplication of NNLS estimated coefficients (matrix *B*) and CIBERSORT estimated cell-type proportions (matrix *p*). The modeled ‘bulk’ expression (matrix *L*) was compared with the ground truth bulk expression (matrix *l*). A linear regression model was used (R function lm) to evaluate the correlation of expression between modeled and ground truth bulk data for each gene (resulting in *P*-value and *R*^2^). Pearson correlation coefficients were calculated using R function cor.test for each sample (Supplementary Figure S3B and Supplementary Table S8).

For circRNAs, modeled ‘bulk’ expression (matrix *N*) of every circRNA in every sample was calculated through matrix multiplication of NNLS estimated coefficients (matrix *C*) and CIBERSORT estimated cell-type proportions (matrix *p*). Modeled ‘bulk’ expression (matrix *N*) was then compared with the ground truth bulk expression (matrix *n*). A linear regression model was used (R function lm) to evaluate the correlation of expression between modeled and ground truth bulk data for each circRNA (resulting in *P*-value and *R*^2^). Pearson correlation coefficients were calculated using R function cor.test for each sample (Supplementary Figure S3C and Supplementary Table S7).

Cell-type specific circRNAs with supporting evidence through scRNA-seq studies were downloaded from circSC (83–85). A total of 3843 circRNAs from cancer cell lines and colon tissue from two studies were used in comparison with cell-type specificity prediction from our analyses (ArrayExpress accession code E-MTAB-6072 and GEO accession code GSE51254).

## RESULTS

### RNA-seq of matched patient cohort identifies abundance of circRNAs throughout mCRC progression

**Figure 2.**
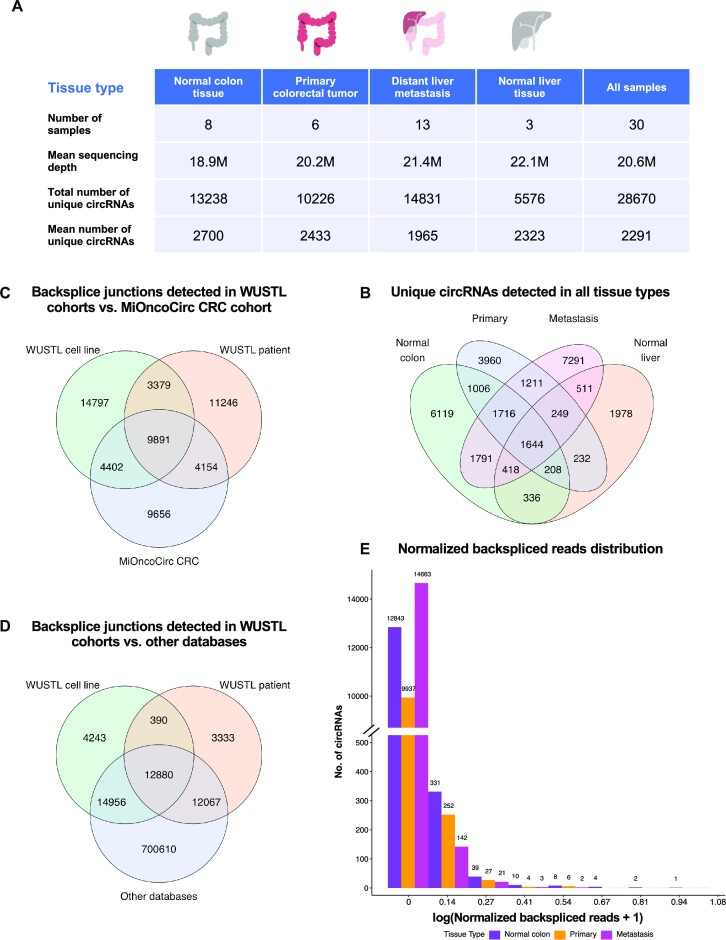
Patient information, sequencing and circRNA detection summary. (**A**) All 30 samples from 14 patients are categorized by tissue types, namely normal colon (normal), primary colorectal tumor (primary), distant liver metastasis (metastasis) and normal liver. For each tissue type, the number of samples, mean sequencing depth, and total and mean numbers of detected circRNAs are tabulated. (**B**) Venn diagram showing the overlap of all 28 760 unique circRNAs detected among different tissue types. (**C**) Venn diagram showing the overlap of unique backsplice junctions detected in WUSTL cell lines, the WUSTL patient cohort and those annotated by MiOncoCirc’s CRC cohort. (**D**) Venn diagram showing the overlap of unique backsplice junctions detected in WUSTL cell lines, the WUSTL patient cohort and those annotated by other databases. (**E**) Bar plot showing backspliced read distribution of circRNAs detected in normal, primary and metastasis samples. Backspliced read numbers were normalized against sequencing depths.

We performed total RNA-seq using 30 samples from 14 stage IV mCRC patients from Washington University in St Louis, termed the WUSTL patient cohort (Figure 1, central column, Figure 2A and Supplementary Table S1). The cohort consisted of 8 normal colon tissue [normal (N)], 6 primary colorectal tumor [primary (P)], 13 distant liver metastasis [metastasis (M)] and 3 adjacent normal liver tissue [normal liver (NL)] samples. The mean sequencing depths among each tissue type were 18.9 (N), 20.2 (P), 21.4 (M) and 22.1 (NL) million reads; the overall mean sequencing depth was 20.6 million reads (Supplementary Table S1).

A total of 28 670 unique circRNAs were detected in all samples, with 13 238 detected in normal, 10 226 in primary, 14 831 in metastasis and 5576 in normal liver. On average, 2291 unique circRNAs were detected in each sample; within each tissue type, the mean numbers of unique circRNAs detected were 2700 in normal, 2433 in primary, 1965 in metastasis and 2323 in normal liver. A total of 4820 unique circRNAs were shared by tumor samples (P, M); 3360 were shared by normal and tumor samples (N, P, M). Overall, 1644 unique circRNAs were shared between all tissue types (Figure 2B).

For downstream analyses of DE and cell-type specificity, RNA-seq reads mapping to linear genes were also quantified (68). A total of 97 847 annotated linear genes were detected, whereby 7176 (7% of expressed genes) were found to have at least one circular isoform. These genes were considered as the parental genes of their corresponding circRNA(s). In general, most detected circRNAs had low expression compared to linear genes. In linear genes, the upper quartile value of mean normalized reads was 1.68 TPM in all samples. On the contrary, the upper quartile value of mean normalized backspliced reads of circRNAs was 0.004 (Figure 2E).

### CircRNA catalog in CRC cell lines

We established a cell line reference catalog by sequencing five common CRC cell lines, including three derived from primary tumors (HCT116, HT29 and SW480) and two from metastatic tumors (SW620 and LoVo), termed WUSTL cell lines (Figure 1, left column). All cell lines underwent cDNA-capture sequencing, as previously described, followed by circRNA quantification (86). The targeted sequencing achieved high on-target rates (97% for all mapped reads and 92% for spliced reads) (64) (Supplementary Table S9).

A total of 32 469 unique circRNAs were detected in all cell lines, originating from 6666 parental genes. A total of 1325 circRNAs were found in common in all cell lines (Supplementary Figure S1A). Out of the 28 670 unique circRNAs from the WUSTL patient cohort, 13 270 (46%) were expressed in at least one cell line sample, while 1289 (4%) were expressed in all cell line samples (Supplementary Figure S1B). All circRNAs detected in cell lines were consolidated into a reference database to serve as a roadmap for future mechanistic and phenotypic validation using cell culture.

### Discovery of novel circRNAs beyond existing annotation

To identify circRNAs that have yet to be characterized in CRC patient samples, we compared our results with the CRC cohort from the pan-cancer circRNA consortium MiOncoCirc (Figure 2C) (74). In our data, 29 422 of all 57 527 backsplice junctions (51%) were not annotated in MiOncoCirc, including 18 176 from WUSTL cell lines and 14 625 from the WUSTL patient cohort. Between cohorts, WUSTL cell lines and MiOncoCirc shared 14 293 backsplice junctions, and the WUSTL patient cohort and MiOncoCirc had 14 045 backsplice junctions in common.

To investigate novel circRNAs unannotated to date, we compared all unique backsplice junctions detected in the WUSTL patient cohort and WUSTL cell lines with the integrative circRNA resource circAtlas encompassing multiple existing circRNA databases, dubbed ‘other databases’ (Figure 2D) (69–77). In our data, 7966 of all 57 527 backsplice junctions (14%) were novel candidates, including 3723 from WUSTL cell lines and 4633 from the WUSTL patient cohort. Between cohorts, WUSTL cell lines and other databases shared 27 836 backsplice junctions, and the WUSTL patient cohort shared 24 947 backsplice junctions with other databases.

It is worth noting that many circRNA databases only characterize unique circRNAs by their backsplice junction coordinates, without taking into consideration possible different isoforms arising from the same backsplice junction. Hence, the true percentage of novel, unannotated circRNAs in our datasets may be >14%.

### DE analysis highlights circRNAs associated with metastasis

**Figure 3.**
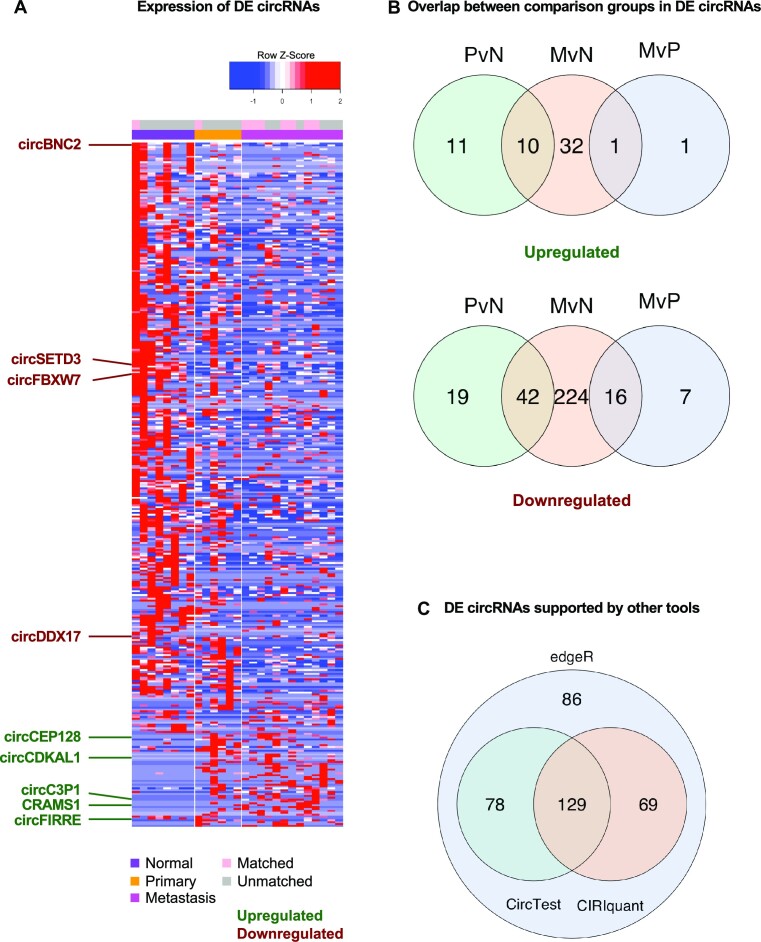
Results of DE analysis of circRNAs in mCRC patients. (**A**) Heatmap of circRNAs differentially expressed in mCRC patient samples. Heatmap color is scaled by row expression *Z*-score. Tissue type and sample match status are color coded in the top row. Dark green text indicates highlight candidates upregulated in mCRC, whereas dark red text indicates highlight candidates downregulated in mCRC. (**B**) Venn diagrams showing the overlap between DE circRNAs in different comparison groups throughout mCRC progression. (**C**) Venn diagrams showing the overlap between DE circRNAs highlighted by different analysis pipelines. The analysis approach used in this study is termed edgeR.

To characterize circRNAs altered in the mCRC progression, we performed DE analysis using edgeR comparing normal, primary and metastatic tissues from the WUSTL patient cohort (Figure 3A). All expressed linear genes and circRNAs were pooled to identify DE circRNAs in CRC patients (78). We identified 362 DE circRNAs in total, of which 55 showed upregulation in at least one of three comparison groups (PvN, MvN and MvP) and 308 showed downregulation (Figure 3B and Supplementary Tables S2 and S3). A total of 69 circRNAs were consistently dysregulated in two or more comparison groups in the same direction, including 11 upregulated and 58 downregulated (Figure 3B and Supplementary Tables S2 and S3). In addition, 319 (88%) DE circRNAs were expressed in at least one cell line sample (Supplementary Table S3).

To provide additional evidence for the DE status of these candidates, we performed two parallel DE tests using CIRIquant and CircTest on the same cohort of circRNAs detected from the WUSTL patient cohort (79,80). Among the 362 DE circRNAs, 276 (76%) showed at least one concurring test, with 129 (36%) validated by both tests (Figure 3C and Supplementary Table S3).

**Figure 4.**
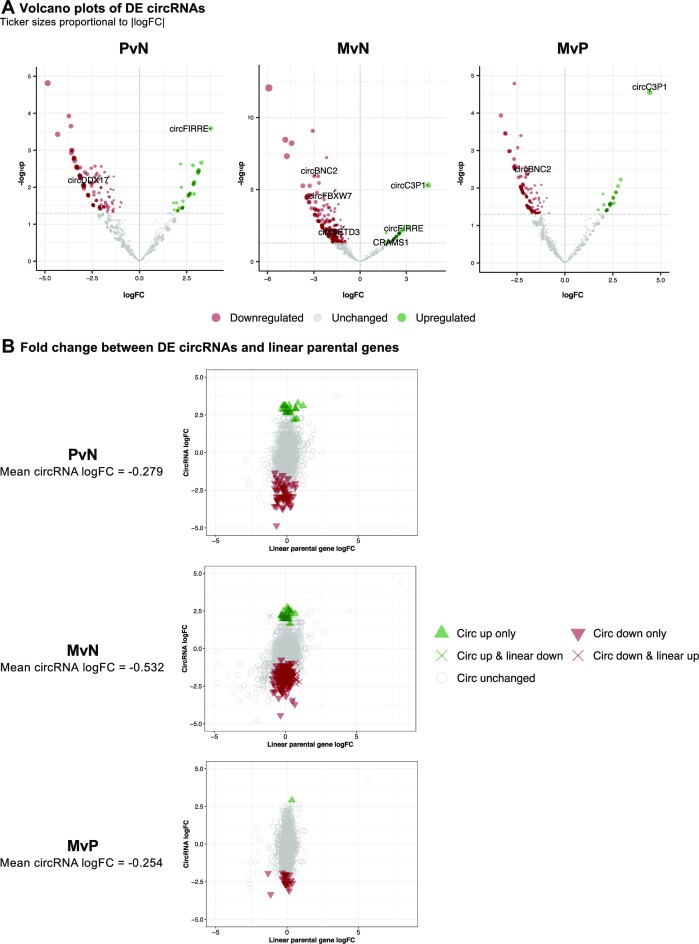
Trends of DE circRNAs across comparison groups. (**A**) Volcano plots of DE circRNAs detected in PvN, MvN and MvP comparison groups from left to right. Ticker sizes are proportional to the absolute value of log FC. CircRNAs with literature evidence and/or were validated by multiple DE analysis pipelines are labeled. (**B**) Correlation plots of log FC of circRNAs and linear genes in PvN, MvN and MvP comparison groups from top to bottom. The mean log FC of circRNAs is indicated next to each panel. The DE status of every circRNA and linear gene pair is represented by the color and shape of the tickers.

Several previously characterized circRNAs that are associated with mCRC or other types of metastatic cancer were shown to be differentially expressed with supporting evidence from at least one other DE test (Figures 3A and C and 4A). This included downregulated candidates circBNC2, circDDX17, circSETD3 and circFBXW7, which have been reported to have tumor suppressing roles by inhibiting migration, invasion and proliferation of metastatic cancers (24,48,87–90). Among the upregulated candidates, circFIRRE and circCEP128 have been shown to promote cancer growth in osteosarcoma and bladder cancer, respectively (91,92). On the contrary, circC3P1, found to be upregulated in metastatic samples, has been reported to be downregulated in hepatocellular carcinoma, warranting further investigation (93).

In general, more circRNAs showed lowered expression in primary or metastatic tumors compared with normal tissues (Figure 4A). For example, DE circRNAs in metastasis compared with normal tissues had a negative mean log FC (−0.532), indicating a global reduction of abundance (Figure 4B, middle panel). A significant portion (29%) of DE circRNAs also had their parental linear genes found to be dysregulated in the same direction (3% both upregulated and 26% both downregulated). Interestingly, a large portion of circRNAs (57%) were found to be dysregulated while their parental linear gene expression was unchanged (53%) or dysregulated in the opposite direction (4%), suggesting alternative mechanisms of circRNA dysregulation (Figure 4B and Supplementary Table S10). A similar trend of reduced abundance was observed when comparing primary tumors with normal tissues, and when comparing metastatic tumors with primary tumors (Figure 4A and B, and Supplementary Table S10).

### Integrative deconvolution models to estimate cell-type specific circRNA expression

**Figure 5.**
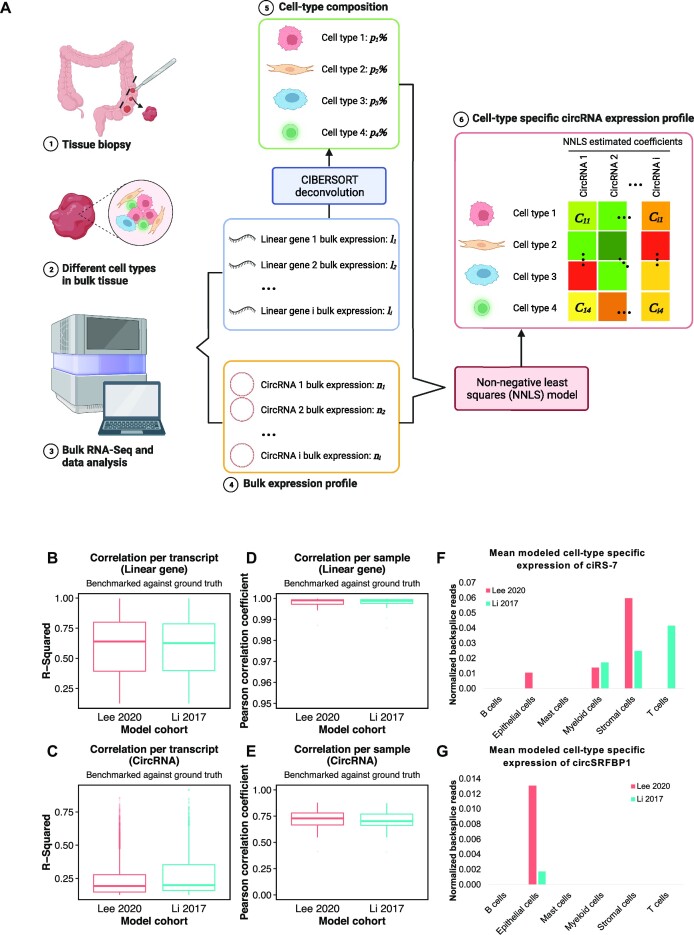
Derivation of cell-type specific expression of circRNAs and NNLS model benchmarking. (**A**) General workflow using one sample. (1, 2) Bulk tissue sample consisting of multiple cell types in the TME was biopsied from an mCRC patient. (3, 4) Bulk expression profiles for both linear genes and circRNAs were built through bulk RNA-seq of tissue samples. (5) Bulk linear gene expression was processed through CIBERSORT using signature matrix based on scRNA-seq cohorts (Lee 2020 or Li 2017) to estimate cell-type composition. (6) Bulk circRNA expression and CIBERSORT estimated cell-type composition were used as inputs in the NNLS model to estimate the cell-type specific circRNA expression profile. (**B**) *R*^2^ values per transcript when comparing modeled ‘bulk’ expression and ground truth bulk expression for each linear gene with *P* < 0.05. In the Lee 2020 model, *R*^2^ ranges from 0.126 to 0.997 with a mean of 0.592; in the Li 2017 model, *R*^2^ ranges from 0.126 to 0.997 with a mean of 0.586. (**C**) *R*^2^ values per transcript when comparing modeled ‘bulk’ expression and ground truth bulk expression for each circRNA with *P* < 0.05. In the Lee 2020 model, *R*^2^ ranges from 0.126 to 0.859 with a mean of 0.226; in the Li 2017 model, *R*^2^ ranges from 0.126 to 0.916 with a mean of 0.266. (**D**) Pearson correlation coefficient values per sample when comparing the linear genes’ modeled ‘bulk’ expression and ground truth bulk expression for each sample. In the Lee 2020 model, Pearson correlation ranges from 0.987 to 1.000 with a mean of 0.998; in the Li 2017 model, Pearson correlation ranges from 0.986 to 1.000 with a mean of 0.998. (**E**) Pearson correlation coefficient values per sample when comparing the circRNAs’ modeled ‘bulk’ expression and ground truth bulk expression for each sample. In the Lee 2020 model, Pearson correlation ranges from 0.413 to 0.878 with a mean of 0.713; in the Li 2017 model, Pearson correlation ranges from 0.411 to 0.873 with a mean of 0.703. (**F**) Cell-type specific expression of ciRS-7 predicted by both models. (**G**) Cell-type specific expression of circSRFBP1 predicted by both models.

To better understand circRNAs dysregulated in CRC in the context of TME, we evaluated their cell-type specific expression in bulk patient RNA-seq. To do so, we leveraged existing patient scRNA-seq data from two independent cohorts, termed the Lee 2020 scRNA-seq cohort (six patients, six cell types, GEO accession code GSE144735) and the Li 2017 scRNA-seq cohort (11 patients, seven cell types, GEO accession code GSE81861), to build signature matrices (Figure 1, right column, Supplementary Figure S2A and B, and Supplementary Table S4) (81,82). Using both datasets in parallel, the bulk linear gene expression of the WUSTL patient samples was deconvolved via CIBERSORT using the signature matrices constructed with the scRNA-seq references (Figure 5A, steps 3–5, Supplementary Figure S2A and B, and Supplementary Table S5) (62).

The resulting cell-type proportion estimates from CIBERSORT were then used to train an NNLS statistical model to estimate the cell-type specific expression value of circRNAs based on their bulk expression (in normalized backspliced reads) values (Figure 5A, step 6). Two models were constructed for both scRNA-seq references, termed the Lee 2020 model and the Li 2017 model. For benchmarking purposes, both models were also run to estimate cell-type specific linear gene expression values.

Both the Lee 2020 model and the Li 2017 model achieved comparable accuracy in recapturing the ground truth expression per transcript, as well as per sample. In general, both models performed better in estimating linear gene expression than circRNA expression. When comparing between transcripts, a higher percentage of linear genes (*P* < 0.05) was predicted by the Lee 2020 model (93%) than by the Li 2017 model (90%). The percentage of circRNAs (*P* < 0.05) from the Lee 2020 model (55%) was also greater than that from the Li 2017 model (46%). There was an overall increase in the linear regression *R*^2^ values in the Lee 2020 model as compared to the Li 2017 model in linear genes with significant *P*-values, with *R*^2^ ranging from 0.126 to 0.997 with a mean of 0.592 in the Lee 2020 model and *R*^2^ ranging from 0.126 to 0.997 with a mean of 0.586 in the Li 2017 model (Figure 5B and Supplementary Table S6). In circRNAs, however, the Li 2017 model performed better in recapturing bulk circRNA expression, with Lee 2020’s *R*^2^ ranging from 0.126 to 0.859 with a mean of 0.226 and Li 2017’s *R*^2^ ranging from 0.126 to 0.916 with a mean of 0.266 for circRNAs with significant *P*-values (Figure 5C and Supplementary Table S6).

When comparing between samples, both models yielded robust real bulk expression estimations of both linear genes and circRNAs, exemplified by all samples having *P* < 0.05 in the association test. In linear genes, the two models performed nearly identically, with the Pearson correlation coefficients ranging from 0.987 to 1.000 with a mean of 0.998 in the Lee 2020 model and ranging from 0.986 to 1.000 with a mean of 0.998 in the Li 2017 model (Figure 5D and Supplementary Table S8). Likewise, in circRNAs, the Lee 2020 model produced Pearson correlation coefficients ranging from 0.413 to 0.878 with a mean of 0.713, which was highly similar to the Pearson correlation coefficients in the Li 2017 model, ranging from 0.411 to 0.873 with a mean of 0.703 (Figure 5E and Supplementary Table S7).

We also investigated the cell-type specific expression of ciRS-7 as a positive control and found evidence corroborating Kristensen *et al.*’s conclusion that ciRS-7 is only found in stromal cells and is absent in CRC epithelial cells (18). The NNLS model trained using the Lee 2020 data estimated ciRS-7 to have the highest expression in stromal cells and very low expression in epithelial cells, whereas the model trained using the Li 2017 data estimated ciRS-7 to have high abundance in endothelial cells, a subtype of stromal cells, and zero expression in epithelial cells (Figure 5F). Interestingly, only 17% of all detected circRNAs were estimated to be expressed in epithelial cells, with 7% shared between both models, 6% in the Lee 2020 model only and 4% in the Li 2017 model only. Only 167 DE (46%) candidates were predicted to be expressed in epithelial cells, warranting further study of the exact mechanisms of their dysregulation in cancer (Supplementary Table S6).

### NNLS model results reveal cell-type specific circRNAs in CRC TME

**Figure 6.**
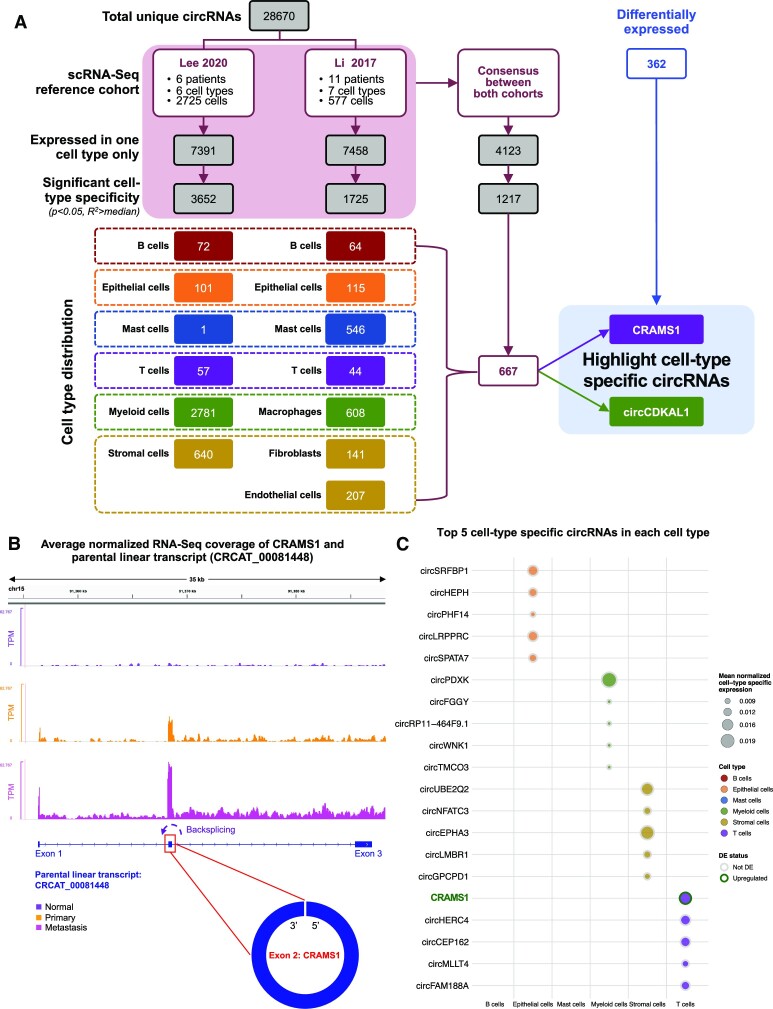
Results of cell-type specific circRNA expression predicted by NNLS models. (**A**) Overview of selection criteria for cell-type specific circRNAs and their cell-type distribution. From the NNLS prediction of both the Lee 2020 and Li 2017 scRNA-seq cohorts, circRNAs are considered significantly cell-type specific if they are expressed in one cell type only and pass two filters: (1) *P* < 0.05 for its NNLS prediction and (2) *R*^2^ is greater than the median *R*^2^ values of all circRNAs. CircRNAs shared by predictions from both models are considered consensus circRNAs. Cell-type specific circRNAs are then separated by the cell type they are expressed in. *Note*: When deciding consensus between models, myeloid cells (the Lee 2020 model) and macrophages (the Li 2017 model) are considered the same cell type; stromal cells (the Lee 2020 model) and fibroblasts and endothelial cells (the Li 2017 model) are considered the same cell type. Overall, the consensus shows that 667 circRNAs are cell-type specific in the same cell type as predicted by both models. When compared with DE circRNAs, circCDKAL1 is specific to myeloid cells, while CRAMS1 is specific to T cells. (**B**) Schematic representation of novel circRNA CRAMS1 in mCRC. The full-length parental lncRNA CRCAT_00081448 is shown below in blue, with its exon 2 constituting CRAMS1. Bar charts show average normalized RNA-seq coverage across normal, primary and metastasis samples in the WUSTL patient cohort. (**C**) Circle plot showing the top 5 cell-type specific circRNAs in each cell type from the consensus between the Lee 2020 and Li 2017 models ranked by *P*-values from lowest to highest. CircRNAs that are specific to the same cell type are displayed in a consecutive order. The size of the circle represents the mean normalized expression of the circRNA among all samples. CRAMS1 is highlighted by dark green to indicate its upregulated DE status.

Based on the Lee 2020 model predictions, 7391 out of 28 670 (26%) unique circRNAs only showed expression in one cell type, while the Li 2017 model predicted 7458 (26%). The consensus between both models showed 4123 (14%) circRNAs with exclusive expression in a single cell type. To select those with significant cell-type specificity, we required the following criteria: *P* < 0.05 and *R*^2^ above the median *R*^2^ of all circRNAs in NNLS estimates. This narrowed the number of cell-type specific circRNAs to 3652 (13%) in the Lee 2020 model and 1725 (6%) in the Li 2017 model, and a consensus of 1217 (4%) between models (Figure 6A). To further consolidate the cell-type specific circRNAs, we required a circRNA to show cell-type specificity in the same cell type in both models. Given the differences in cell types annotated by the Lee 2020 and Li 2017 models, we considered macrophages as a subtype of myeloid cells, and fibroblasts and endothelial cells as subtypes of stromal cells. A total of 667 candidates fulfilled the criteria as consensus cell-type specific circRNAs (Figure 6A).

While our cell-type specificity results were derived computationally from bulk RNA-seq data, we compared our predictions with cell-type specific circRNAs reported in the recently published database circSC (83). Sixteen circRNAs predicted by our pipeline to be cell-type specific were present in circSC, and four (25%) were found to be specific in the same cell types as shown by scRNA-seq studies. For example, circSRFBP1, the candidate with the highest specificity to epithelial cells based on NNLS *P*-values, was reported to be specific to CRC cells (Figure 5G and Figure 6C, Supplementary Figure 4D and Supplementary Table S11).

When compared with DE circRNAs, two upregulated circRNAs, CRAMS1 and circCDKAL1, were estimated to be expressed in one cell type only (Figure 6A–C and Supplementary Figure S4A–C). CircCDKAL1 was among the consensus of both NNLS models to be exclusively expressed in myeloid cells, while CRAMS1 expression was exclusive to T cells (Figure 6A, Supplementary Figure S4A and B, and Supplementary Table S6). Markedly, CRAMS1 was predicted to have the highest specificity to T cells based on NNLS *P*-values (Figure 6C).

Many DE circRNAs have ample literature evidence supporting their dysregulation in CRC or other cancers (Figure 3A). However, through our cell-type specificity analysis, neither circCDKAL1 nor CRAMS1 has been reported to be associated with metastasis. When examining the linear parental genes, CDKAL1 is known to be implicated in endometrial cancer tumorigenesis (94). On the other hand, CRAMS1’s parental transcript CRCAT_00081448 is a novel lncRNA characterized by our lab’s *de novo* assembly in mCRC patient samples, however, without previous report on its role in mCRC progression (Figure 6B) (65). The scarcity of existing evidence necessitates the need for future studies of these dysregulated, TME cell-type specific candidates in CRC.

## DISCUSSION

Our study has addressed many limitations of existing CRC research. To date, there has not been a comprehensive, spatial expression profile for circRNAs in mCRC. For example, the pan-cancer database of expressed circRNAs across cancers, MiOncoCirc, consists of 868 patient samples, of which 217 (25%) are from prostate cancer, whereas only 13 (1.5%) samples are from CRC. Further, since these CRC samples were not curated from adjacent sites of normal, primary and metastatic tissues from the same patients, it proved challenging to systematically identify dysregulated circRNAs throughout mCRC progression (74). Our unique matched normal, primary and metastasis samples from mCRC patient cohorts allowed us to discover circRNAs altered throughout mCRC progression. With our integrated computational approach, we not only discovered circRNAs altered in mCRC, but also elucidated their potential cell-type specificity. We envision that these novel findings will serve as a resource for future mechanistic and functional studies in circRNAs in relation to the TME of CRC. Our computational pipeline can be adapted to study circRNAs in other tissues and diseases as well, serving as a versatile tool. In addition, we established an extensive collection of circRNAs in CRC cell lines that can be used to delve into the oncogenic roles of candidate circRNAs identified above (Supplementary Figure S1A and B, and Supplementary Table S9).

We performed transcriptome profiling of a matched cohort of mCRC patients and characterized CRAMS. We detected 28 670 unique circRNAs, 51% of which were isoforms unannotated in MiOncoCirc (Figure 2C). Through DE analysis, we highlighted 362 CRAMS that are DE circRNAs throughout mCRC progression (Figure 3A and B, and Supplementary Tables S2 and S3). Three-quarters of CRAMS were supported by orthogonal validation in spite of the intrinsic differences between CIRIquant, CircTest and our DE analysis approach. Despite providing the CIRCexplorer2 detection output as guidance, CIRIquant is based on different alignment and read assembly software than that in our pipeline, resulting in slightly different read counts for the downstream DE analysis (79). On the other hand, CircTest solely relies on the parental linear transcripts that have corresponding circRNAs, thereby omitting a large number of linear genes (90 671 genes, 93% of all expressed genes) and affecting the scaling and normalization of the read counts during the DE analysis (80). We wish to use these supporting test results as robust evidence for the CRAMS identified and emphasize candidates that have high confidence when validated by more than one pipeline.

A vast majority of DE circRNAs were downregulated in primary or metastatic tumors, which could be attributed to the faster cell proliferation diluting their global abundance as the cancer progresses, or increased export to exosomes due to their high stability (14,74). Comparing circRNAs and their parental linear genes showed that only approximately one-third of the DE circRNAs were co-dysregulated with their parental genes, whereas the rest of them had parental genes that were unchanged or even dysregulated in the opposite direction (Figure 4B). This suggests while circRNA expression could be attributed to parental gene regulation, it could also be independent of their parental gene in some cases. Therefore, further investigation is needed to precisely define how circRNAs are regulated in mCRC.

Identifying the spatial origin and cell-type specific expression of circRNAs is paramount to understanding their biological roles as potential TME regulators. To address the lack of cell-type deconvolution methods involving circRNAs, we devised a unique computational approach to identify cell-type specific circRNAs associated with the TME of CRC. By combining deconvolution analysis using two published scRNA-seq datasets and building an NNLS statistical model, we discovered that 667 circRNAs were exclusively expressed in one cell type only. Our results were congruent with earlier findings by highlighting known cell-type specific circRNAs such as ciRS-7 being exclusive to stromal cells (Figure 5F) (18). In particular, four predicted cell-type specific candidates, such as the epithelial cell-specific circSRFBP1, had supporting scRNA-seq evidence from circSC despite the highly heterogeneous nature of the datasets included in circSC (Figure 5G). Many studies in circSC focus on other cancers or diseases and are heavily skewed toward hematopoietic cell types that are absent in solid tumors like CRC, and only two out of 171 datasets from circSC contained cell types relevant to this comparison. Moreover, there is a lack of stromal and myeloid cell presence in these scRNA-seq datasets. Hence, we believe that our findings reported in TMECircDB will serve as valuable knowledge for cell-type specific circRNAs that are unique to the TME of metastatic CRC.

Through benchmarking, we showed that the NNLS models derived from both the Lee 2020 and Li 2017 scRNA-seq cohorts achieved highly robust performance in predicting cell-type specific expression by accurately recapturing bulk expression of linear genes. When applied to circRNAs, both models performed comparably, but only successfully recaptured the bulk expression in approximately half of the circRNAs. In the circRNAs that yielded statistically significant predictions, the overall *R*^2^ range was similar to that of the linear genes but with lower maximum *R*^2^ values (Figure 5B and C). However, the distribution of *R*^2^ values in circRNAs was clustered below 0.2 with a large number of outliers in the upper end of the *R*^2^ spectra (Figure 5C). In linear genes, most *R*^2^ values were clustered above 0.6 and there were no outliers in either model (Figure 5B). This is likely due to the overall low abundance of circRNAs as compared to linear genes, hence providing less information about their expression profiles for the statistical models to estimate cell-type specificity (Figure 2E). Despite this caveat, our analysis predicted 1217 consensus circRNAs with statistically significant cell-type specific expression, 667 of which were cell-type specific in the same cell type as agreed upon by both models. This suggests that these consensus candidates were detected with high confidence even when the majority of *R*^2^ values were low. Moreover, when comparing between samples, the Pearson correlation coefficients in linear genes were close to 1 in both models, showing nearly perfect correlation (Figure 5D). In circRNAs, the median Pearson correlation coefficients also showed high correlation per sample, albeit lower than that in linear genes (Figure 5E). This shows that both models performed robustly in recapturing the ground truth expression of circRNAs in each sample.

Among the DE and cell-type specific circRNAs, we highlighted several candidates that showed promising association with CRC metastasis (Figure 3A). With a global downregulation trend of circRNAs in cancer, the upregulated circFIRRE and CRAMS1 are particularly worth investigating further. While circFIRRE’s parental transcript FIRRE was also upregulated in primary and metastasis samples, suggesting parental gene regulation contribution, CRAMS1 was upregulated independent of its parental transcript CRCAT_00081448, which was unchanged in mCRC (Figure 6B). Furthermore, CRAMS1 displayed cell-type specificity in T cells, which could be a result of increased immune response in cancer (Supplementary Figure S4B). In addition, certain circRNAs could be involved in the infiltration of certain myeloid populations such as tumor-associated macrophages, which are known to promote metastasis, as suggested by Song *et al.* (17). Hence, we speculate that the myeloid cell-specific upregulated circRNA circCDKAL1 could be involved in facilitating the crosstalk of the CRC TME (Supplementary Figure S4A and C). As these candidates have little to no literature support in CRC research, there is substantial room for more experimental evidence to emerge. While our study provides an insightful resource, further experiment validation is required to show the mechanism and exact function of the CRAMS we have reported.

## CONCLUSION

This research will have a broad overall impact on the field of RNA tumor biology by using human patients to provide a comprehensive resource for defining the landscape of circRNAs altered during metastatic CRC progression. Our computational methodology also enabled further deconvolution of circRNA expression into tumor microenvironment cell types that can guide future mechanistic studies of circRNAs contributing to mCRC.

## DATA AVAILABILITY

The cell-type specific expression data of identified circRNAs are available at the TMECircDB resource: https://www.maherlab.com/tmecircdb-overview. The data analysis pipeline and related scripts are available at Zenodo (https://doi.org/10.5281/zenodo.7884187) and GitHub (https://github.com/ChrisMaherLab/TMECircDB). The RNA-seq data generated in this study (WUSTL patient cohort and WUSTL cell lines) have been deposited in the NCBI Gene Expression Omnibus database under the accession code GSE221240. The Lee 2020 scRNA-seq data referenced during the study are available in a public repository from the NCBI Gene Expression Omnibus under the accession code GSE144735. The Li 2017 scRNA-seq data referenced during the study are available in a public repository from the NCBI Gene Expression Omnibus under the accession code GSE81861. All the other data supporting the findings of this study are available within the article and its Supplementary Data and from the corresponding author upon reasonable request.

## Supplementary Material

zcad021_Supplemental_Files

## References

[1] Bray F., Ferlay J., Soerjomataram I., Siegel R.L., Torre L.A., Jemal A. Global cancer statistics 2018: GLOBOCAN estimates of incidence and mortality worldwide for 36 cancers in 185 countries. CA Cancer J. Clin. 2018; 68:394–424. 30207593 10.3322/caac.21492

[2] American Cancer Society Colorectal Cancer Facts & Figures 2017–2019. 2017; Atlanta, GA American Cancer Society.

[3] Chaffer C.L., Weinberg R.A. A perspective on cancer cell metastasis. Science. 2011; 331:1559–1564. 21436443 10.1126/science.1203543

[4] Zeng Y., Xu Y., Shu R., Sun L., Tian Y., Shi C., Zheng Z., Wang K., Luo H. Altered expression profiles of circular RNA in colorectal cancer tissues from patients with lung metastasis. Int. J. Mol. Med. 2017; 40:1818–1828. 29039473 10.3892/ijmm.2017.3189 PMC5716445

[5] Siegel R.L., Miller K.D., Jemal A. Cancer statistics, 2019. CA Cancer J. Clin. 2019; 69:7–34. 30620402 10.3322/caac.21551

[6] Cho K.R., Vogelstein B. Genetic alterations in the adenoma–carcinoma sequence. Cancer. 1992; 70:1727–1731. 1516027 10.1002/1097-0142(19920915)70:4+<1727::aid-cncr2820701613>3.0.co;2-p

[7] Jeck W.R., Sharpless N.E. Detecting and characterizing circular RNAs. Nat. Biotechnol. 2014; 32:453–461. 24811520 10.1038/nbt.2890 PMC4121655

[8] Bose R., Ain R. Regulation of transcription by circular RNAs. Adv. Exp. Med. Biol. 2018; 1087:81–94. 30259359 10.1007/978-981-13-1426-1_7

[9] Xu T., Wu J., Han P., Zhao Z., Song X. Circular RNA expression profiles and features in human tissues: a study using RNA-seq data. BMC Genomics. 2017; 18:680. 28984197 10.1186/s12864-017-4029-3 PMC5629547

[10] Jeck W.R., Sorrentino J.A., Wang K., Slevin M.K., Burd C.E., Liu J., Marzluff W.F., Sharpless N.E. Circular RNAs are abundant, conserved, and associated with ALU repeats. RNA. 2013; 19:141–157. 23249747 10.1261/rna.035667.112 PMC3543092

[11] Holdt L.M., Kohlmaier A., Teupser D. Circular RNAs as therapeutic agents and targets. Front. Physiol. 2018; 9:1262. 30356745 10.3389/fphys.2018.01262 PMC6189416

[12] Lasda E., Parker R. Circular RNAs: diversity of form and function. RNA. 2014; 20:1829–1842. 25404635 10.1261/rna.047126.114 PMC4238349

[13] Salzman J. Circular RNA expression: its potential regulation and function. Trends Genet. 2016; 32:309–316. 27050930 10.1016/j.tig.2016.03.002 PMC4948998

[14] Dou Y., Cha D.J., Franklin J.L., Higginbotham J.N., Jeppesen D.K., Weaver A.M., Prasad N., Levy S., Coffey R.J., Patton J.G. et al. Circular RNAs are down-regulated in KRAS mutant colon cancer cells and can be transferred to exosomes. Sci. Rep. 2016; 6:37982. 27892494 10.1038/srep37982 PMC5125100

[15] Bach D.H., Lee S.K., Sood A.K. Circular RNAs in cancer. Mol. Ther. Nucleic Acids. 2019; 16:118–129. 30861414 10.1016/j.omtn.2019.02.005 PMC6411617

[16] Liang D., Tatomer D.C., Luo Z., Wu H., Yang L., Chen L.L., Cherry S., Wilusz J.E. The output of protein-coding genes shifts to circular RNAs when the pre-mRNA processing machinery is limiting. Mol. Cell. 2017; 68:940–954. 29174924 10.1016/j.molcel.2017.10.034 PMC5728686

[17] Song H., Liu Q., Liao Q. Circular RNA and tumor microenvironment. Cancer Cell Int. 2020; 20:211. 32518520 10.1186/s12935-020-01301-z PMC7268656

[18] Kristensen L.S., Ebbesen K.K., Sokol M., Jakobsen T., Korsgaard U., Eriksen A.C., Hansen T.B., Kjems J., Hager H. Spatial expression analyses of the putative oncogene ciRS-7 in cancer reshape the microRNA sponge theory. Nat. Commun. 2020; 11:4551. 32917870 10.1038/s41467-020-18355-2 PMC7486402

[19] Bachmayr-Heyda A., Reiner A.T., Auer K., Sukhbaatar N., Aust S., Bachleitner-Hofmann T., Mesteri I., Grunt T.W., Zeillinger R., Pils D. Correlation of circular RNA abundance with proliferation—exemplified with colorectal and ovarian cancer, idiopathic lung fibrosis, and normal human tissues. Sci. Rep. 2015; 5:8057. 25624062 10.1038/srep08057 PMC4306919

[20] Chen S., Zhang L., Su Y., Zhang X. Screening potential biomarkers for colorectal cancer based on circular RNA chips. Oncol. Rep. 2018; 39:2499–2512. 29658599 10.3892/or.2018.6372 PMC5983920

[21] Jiang W., Zhang X., Chu Q., Lu S., Zhou L., Lu X., Liu C., Mao L., Ye C., Timko M.P. et al. The circular RNA profiles of colorectal tumor metastatic cells. Front. Genet. 2018; 9:34. 29479369 10.3389/fgene.2018.00034 PMC5811837

[22] Ju H.Q., Zhao Q., Wang F., Lan P., Wang Z., Zuo Z.X., Wu Q.N., Fan X.J., Mo H.Y., Chen L. et al. A circRNA signature predicts postoperative recurrence in stage II/III colon cancer. EMBO Mol. Med. 2019; 11:e10168. 31475771 10.15252/emmm.201810168 PMC6783650

[23] Li H., Jin X., Liu B., Zhang P., Chen W., Li Q. CircRNA CBL.11 suppresses cell proliferation by sponging miR-6778-5p in colorectal cancer. BMC Cancer. 2019; 19:826. 31438886 10.1186/s12885-019-6017-2 PMC6704711

[24] Li X.N., Wang Z.J., Ye C.X., Zhao B.C., Li Z.L., Yang Y. RNA sequencing reveals the expression profiles of circRNA and indicates that circDDX17 acts as a tumor suppressor in colorectal cancer. J. Exp. Clin. Cancer Res. 2018; 37:325. 30591054 10.1186/s13046-018-1006-x PMC6307166

[25] Tian Y., Xu Y., Wang H., Shu R., Sun L., Zeng Y., Gong F., Lei Y., Wang K., Luo H. Comprehensive analysis of microarray expression profiles of circRNAs and lncRNAs with associated co-expression networks in human colorectal cancer. Funct. Integr. Genomics. 2019; 19:311–327. 30446877 10.1007/s10142-018-0641-9 PMC6394731

[26] Xiong W., Ai Y.Q., Li Y.F., Ye Q., Chen Z.T., Qin J.Y., Liu Q.Y., Wang H., Ju Y.H., Li W.H. Microarray analysis of circular RNA expression profile associated with 5-fluorouracil-based chemoradiation resistance in colorectal cancer cells. Biomed. Res. Int. 2017; 2017:8421614. 28656150 10.1155/2017/8421614 PMC5471554

[27] Xu H., Wang C., Song H., Xu Y., Ji G. RNA-seq profiling of circular RNAs in human colorectal cancer liver metastasis and the potential biomarkers. Mol. Cancer. 2019; 18:8. 30630466 10.1186/s12943-018-0932-8 PMC6327571

[28] Zhang J., Liu H., Zhao P., Zhou H., Mao T. Has_circ_0055625 from circRNA profile increases colon cancer cell growth by sponging miR-106b-5p. J. Cell. Biochem. 2019; 120:3027–3037. 30520100 10.1002/jcb.27355

[29] Zhang P., Zuo Z., Shang W., Wu A., Bi R., Wu J., Li S., Sun X., Jiang L. Identification of differentially expressed circular RNAs in human colorectal cancer. Tumour Biol. 2017; 39:1010428317694546. 28349836 10.1177/1010428317694546

[30] Zhang Z., Song N., Wang Y., Zhong J., Gu T., Yang L., Shen X., Li Y., Yang X., Liu X. et al. Analysis of differentially expressed circular RNAs for the identification of a coexpression RNA network and signature in colorectal cancer. J. Cell. Biochem. 2019; 120:6409–6419. 30320923 10.1002/jcb.27928

[31] Zheng X., Chen L., Zhou Y., Wang Q., Zheng Z., Xu B., Wu C., Zhou Q., Hu W., Jiang J. A novel protein encoded by a circular RNA circPPP1R12A promotes tumor pathogenesis and metastasis of colon cancer via Hippo-YAP signaling. Mol. Cancer. 2019; 18:47. 30925892 10.1186/s12943-019-1010-6 PMC6440158

[32] Zhu M., Xu Y., Chen Y., Yan F. Circular BANP, an upregulated circular RNA that modulates cell proliferation in colorectal cancer. Biomed. Pharmacother. 2017; 88:138–144. 28103507 10.1016/j.biopha.2016.12.097

[33] Zhuo F., Lin H., Chen Z., Huang Z., Hu J. The expression profile and clinical significance of circRNA0003906 in colorectal cancer. OncoTargets Ther. 2017; 10:5187–5193. 10.2147/OTT.S147378 PMC5661852 29123417

[34] Bian L., Zhi X., Ma L., Zhang J., Chen P., Sun S., Li J., Sun Y., Qin J. Hsa_circRNA_103809 regulated the cell proliferation and migration in colorectal cancer via miR-532-3p/FOXO4 axis. Biochem. Biophys. Res. Commun. 2018; 505:346–352. 30249393 10.1016/j.bbrc.2018.09.073

[35] Chen Y., Yang F., Fang E., Xiao W., Mei H., Li H., Li D., Song H., Wang J., Hong M. et al. Circular RNA circAGO2 drives cancer progression through facilitating HuR-repressed functions of AGO2–miRNA complexes. Cell Death Differ. 2019; 26:1346–1364. 30341421 10.1038/s41418-018-0220-6 PMC6748083

[36] Fang G., Ye B.L., Hu B.R., Ruan X.J., Shi Y.X. CircRNA_100290 promotes colorectal cancer progression through miR-516b-induced downregulation of FZD4 expression and Wnt/β-catenin signaling. Biochem. Biophys. Res. Commun. 2018; 504:184–189. 30173892 10.1016/j.bbrc.2018.08.152

[37] Guo J.N., Li J., Zhu C.L., Feng W.T., Shao J.X., Wan L., Huang M.D., He J.D. Comprehensive profile of differentially expressed circular RNAs reveals that hsa_circ_0000069 is upregulated and promotes cell proliferation, migration, and invasion in colorectal cancer. OncoTargets Ther. 2016; 9:7451–7458. 10.2147/OTT.S123220 PMC5158168 28003761

[38] He J.H., Li Y.G., Han Z.P., Zhou J.B., Chen W.M., Lv Y.B., He M.L., Zuo J.D., Zheng L. The CircRNA-ACAP2/hsa-miR-21-5p/Tiam1 regulatory feedback circuit affects the proliferation, migration, and invasion of colon cancer SW480 cells. Cell. Physiol. Biochem. 2018; 49:1539–1550. 30212824 10.1159/000493457

[39] Huang G., Zhu H., Shi Y., Wu W., Cai H., Chen X. cir-ITCH plays an inhibitory role in colorectal cancer by regulating the Wnt/β-catenin pathway. PLoS One. 2015; 10:e0131225. 26110611 10.1371/journal.pone.0131225 PMC4482251

[40] Ji W., Qiu C., Wang M., Mao N., Wu S., Dai Y. Hsa_circ_0001649: a circular RNA and potential novel biomarker for colorectal cancer. Biochem. Biophys. Res. Commun. 2018; 497:122–126. 29421663 10.1016/j.bbrc.2018.02.036

[41] Jin C., Wang A., Liu L., Wang G., Li G. Hsa_circ_0136666 promotes the proliferation and invasion of colorectal cancer through miR-136/SH2B1 axis. J. Cell. Physiol. 2019; 234:7247–7256. 30370521 10.1002/jcp.27482

[42] Jin Y., Yu L.L., Zhang B., Liu C.F., Chen Y. Circular RNA hsa_circ_0000523 regulates the proliferation and apoptosis of colorectal cancer cells as miRNA sponge. Braz. J. Med. Biol. Res. 2018; 51:e7811. 30403259 10.1590/1414-431X20187811 PMC6233523

[43] Li J., Ni S., Zhou C., Ye M. The expression profile and clinical application potential of hsa_circ_0000711 in colorectal cancer. Cancer Manag. Res. 2018; 10:2777–2784. 30147374 10.2147/CMAR.S172388 PMC6103302

[44] Li X., Wang J., Zhang C., Lin C., Zhang J., Zhang W., Lu Y., Zheng L. Circular RNA circITGA7 inhibits colorectal cancer growth and metastasis by modulating the Ras pathway and upregulating transcription of its host gene ITGA7. J. Pathol. 2018; 246:166–179. 29943828 10.1002/path.5125

[45] Min L., Wang H., Zeng Y. CircRNA_104916 regulates migration, apoptosis and epithelial–mesenchymal transition in colon cancer cells. Front. Biosci. (Landmark Ed.). 2019; 24:819–832. 30844715 10.2741/4753

[46] Tang W., Ji M., He G., Yang L., Niu Z., Jian M., Wei Y., Ren L., Xu J. Silencing CDR1as inhibits colorectal cancer progression through regulating microRNA-7. OncoTargets Ther. 2017; 10:2045–2056. 10.2147/OTT.S131597 PMC5391170 28435295

[47] Wang F., Wang J., Cao X., Xu L., Chen L. Hsa_circ_0014717 is downregulated in colorectal cancer and inhibits tumor growth by promoting p16 expression. Biomed. Pharmacother. 2018; 98:775–782. 29571246 10.1016/j.biopha.2018.01.015

[48] Wang J., Li X., Lu L., He L., Hu H., Xu Z. Circular RNA hsa_circ_0000567 can be used as a promising diagnostic biomarker for human colorectal cancer. J. Clin. Lab. Anal. 2018; 32:e22379. 29333615 10.1002/jcla.22379 PMC6817158

[49] Wang X., Zhang Y., Huang L., Zhang J., Pan F., Li B., Yan Y., Jia B., Liu H., Li S. et al. Decreased expression of hsa_circ_001988 in colorectal cancer and its clinical significances. Int. J. Clin. Exp. Pathol. 2015; 8:16020–16025. 26884878 PMC4730091

[50] Weng W., Wei Q., Toden S., Yoshida K., Nagasaka T., Fujiwara T., Cai S., Qin H., Ma Y., Goel A. Circular RNA ciRS-7-A promising prognostic biomarker and a potential therapeutic target in colorectal cancer. Clin. Cancer Res. 2017; 23:3918–3928. 28174233 10.1158/1078-0432.CCR-16-2541 PMC5511556

[51] Xie H., Ren X., Xin S., Lan X., Lu G., Lin Y., Yang S., Zeng Z., Liao W., Ding Y.Q. et al. Emerging roles of circRNA_001569 targeting miR-145 in the proliferation and invasion of colorectal cancer. OncoTargets Ther. 2016; 7:26680–26691. 10.18632/oncotarget.8589 PMC5042007 27058418

[52] Xu X.W., Zheng B.A., Hu Z.M., Qian Z.Y., Huang C.J., Liu X.Q., Wu W.D. Circular RNA hsa_circ_000984 promotes colon cancer growth and metastasis by sponging miR-106b. OncoTargets Ther. 2017; 8:91674–91683. 10.18632/oncotarget.21748 PMC5710956 29207676

[53] Yong W., Zhuoqi X., Baocheng W., Dongsheng Z., Chuan Z., Yueming S. Hsa_circ_0071589 promotes carcinogenesis via the miR-600/EZH2 axis in colorectal cancer. Biomed. Pharmacother. 2018; 102:1188–1194. 29710537 10.1016/j.biopha.2018.03.085

[54] Yuan Y., Liu W., Zhang Y., Sun S. CircRNA circ_0026344 as a prognostic biomarker suppresses colorectal cancer progression via microRNA-21 and microRNA-31. Biochem. Biophys. Res. Commun. 2018; 503:870–875. 29928882 10.1016/j.bbrc.2018.06.089

[55] Zeng K., Chen X., Xu M., Liu X., Hu X., Xu T., Sun H., Pan Y., He B., Wang S. CircHIPK3 promotes colorectal cancer growth and metastasis by sponging miR-7. Cell Death Dis. 2018; 9:417. 29549306 10.1038/s41419-018-0454-8 PMC5856798

[56] Zhang X.L., Xu L.L., Wang F. Hsa_circ_0020397 regulates colorectal cancer cell viability, apoptosis and invasion by promoting the expression of the miR-138 targets TERT and PD-L1. Cell Biol. Int. 2017; 41:1056–1064. 28707774 10.1002/cbin.10826

[57] Zhong D., Li P., Gong P.Y. Hsa_circ_0005075 promotes the proliferation and invasion of colorectal cancer cells. Int. J. Biol. Markers. 2019; 34:284–291. 31476947 10.1177/1724600819872765

[58] Zhang R., Xu J., Zhao J., Wang X. Silencing of hsa_circ_0007534 suppresses proliferation and induces apoptosis in colorectal cancer cells. Riv. Eur. Sci. Med. Farmacol. 2018; 22:118–126. 10.26355/eurrev_201801_14108 29364478

[59] Cancer Genome Atlas Network Comprehensive molecular characterization of human colon and rectal cancer. Nature. 2012; 487:330–337. 22810696 10.1038/nature11252 PMC3401966

[60] Fan X., Zhang X., Wu X., Guo H., Hu Y., Tang F., Huang Y. Single-cell RNA-seq transcriptome analysis of linear and circular RNAs in mouse preimplantation embryos. Genome Biol. 2015; 16:148. 26201400 10.1186/s13059-015-0706-1 PMC4511241

[61] Newman A.M., Steen C.B., Liu C.L., Gentles A.J., Chaudhuri A.A., Scherer F., Khodadoust M.S., Esfahani M.S., Luca B.A., Steiner D. et al. Determining cell type abundance and expression from bulk tissues with digital cytometry. Nat. Biotechnol. 2019; 37:773–782. 31061481 10.1038/s41587-019-0114-2 PMC6610714

[62] Newman A.M., Liu C.L., Green M.R., Gentles A.J., Feng W., Xu Y., Hoang C.D., Diehn M., Alizadeh A.A. Robust enumeration of cell subsets from tissue expression profiles. Nat. Methods. 2015; 12:453–457. 25822800 10.1038/nmeth.3337 PMC4739640

[63] Lawson C.L., Hanson R.J. Solving Least Squares Problems. Classics in Applied Mathematics. 1995; Philadelphia, PA SIAM.

[64] Quinlan A.R., Hall I.M. BEDTools: a flexible suite of utilities for comparing genomic features. Bioinformatics. 2010; 26:841–842. 20110278 10.1093/bioinformatics/btq033 PMC2832824

[65] Silva-Fisher J.M., Dang H.X., White N.M., Strand M.S., Krasnick B.A., Rozycki E.B., Jeffers G.G.L., Grossman J.G., Highkin M.K., Tang C. et al. Long non-coding RNA RAMS11 promotes metastatic colorectal cancer progression. Nat. Commun. 2020; 11:2156. 32358485 10.1038/s41467-020-15547-8 PMC7195452

[66] Dobin A., Davis C.A., Schlesinger F., Drenkow J., Zaleski C., Jha S., Batut P., Chaisson M., Gingeras T.R. STAR: ultrafast universal RNA-seq aligner. Bioinformatics. 2013; 29:15–21. 23104886 10.1093/bioinformatics/bts635 PMC3530905

[67] Zhang X.O., Dong R., Zhang Y., Zhang J.L., Luo Z., Zhang J., Chen L.L., Yang L. Diverse alternative back-splicing and alternative splicing landscape of circular RNAs. Genome Res. 2016; 26:1277–1287. 27365365 10.1101/gr.202895.115 PMC5052039

[68] Liao Y., Smyth G.K., Shi W. featureCounts: an efficient general purpose program for assigning sequence reads to genomic features. Bioinformatics. 2014; 30:923–930. 24227677 10.1093/bioinformatics/btt656

[69] Wu W., Ji P., Zhao F. CircAtlas: an integrated resource of one million highly accurate circular RNAs from 1070 vertebrate transcriptomes. Genome Biol. 2020; 21:101. 32345360 10.1186/s13059-020-02018-y PMC7187532

[70] Liu M., Wang Q., Shen J., Yang B.B., Ding X. Circbank: a comprehensive database for circRNA with standard nomenclature. RNA Biol. 2019; 16:899–905. 31023147 10.1080/15476286.2019.1600395 PMC6546381

[71] Glažar P., Papavasileiou P., Rajewsky N. circBase: a database for circular RNAs. RNA. 2014; 20:1666–1670. 25234927 10.1261/rna.043687.113 PMC4201819

[72] Dong R., Ma X.K., Li G.W., Yang L. CIRCpedia v2: an updated database for comprehensive circular RNA annotation and expression comparison. Genomics Proteomics Bioinformatics. 2018; 16:226–233. 30172046 10.1016/j.gpb.2018.08.001 PMC6203687

[73] Ruan H., Xiang Y., Ko J., Li S., Jing Y., Zhu X., Ye Y., Zhang Z., Mills T., Feng J. et al. Comprehensive characterization of circular RNAs in ∼1000 human cancer cell lines. Genome Med. 2019; 11:55. 31446897 10.1186/s13073-019-0663-5 PMC6709551

[74] Vo J.N., Cieslik M., Zhang Y., Shukla S., Xiao L., Wu Y.M., Dhanasekaran S.M., Engelke C.G., Cao X., Robinson D.R. et al. The landscape of circular RNA in cancer. Cell. 2019; 176:869–881. 30735636 10.1016/j.cell.2018.12.021 PMC6601354

[75] Zheng L.-L., Li J.-H., Wu J., Sun W.-J., Liu S., Wang Z.-L., Zhou H., Yang J.-H., Qu L.-H. deepBase v2.0: identification, expression, evolution and function of small RNAs, lncRNAs and circular RNAs from deep-sequencing data. Nucleic Acids Res. 2016; 44:D196–D202. 26590255 10.1093/nar/gkv1273 PMC4702900

[76] Xia S., Feng J., Lei L., Hu J., Xia L., Wang J., Xiang Y., Liu L., Zhong S., Han L. et al. Comprehensive characterization of tissue-specific circular RNAs in the human and mouse genomes. Brief. Bioinform. 2017; 18:984–992. 27543790 10.1093/bib/bbw081

[77] Xia S., Feng J., Chen K., Ma Y., Gong J., Cai F., Jin Y., Gao Y., Xia L., Chang H. et al. CSCD: a database for cancer-specific circular RNAs. Nucleic Acids Res. 2018; 46:D925–D929. 29036403 10.1093/nar/gkx863 PMC5753219

[78] Robinson M.D., McCarthy D.J., Smyth G.K. edgeR: a Bioconductor package for differential expression analysis of digital gene expression data. Bioinformatics. 2010; 26:139–140. 19910308 10.1093/bioinformatics/btp616 PMC2796818

[79] Zhang J., Chen S., Yang J., Zhao F. Accurate quantification of circular RNAs identifies extensive circular isoform switching events. Nat. Commun. 2020; 11:90. 31900416 10.1038/s41467-019-13840-9 PMC6941955

[80] Cheng J., Metge F., Dieterich C. Specific identification and quantification of circular RNAs from sequencing data. Bioinformatics. 2016; 32:1094–1096. 26556385 10.1093/bioinformatics/btv656

[81] Lee H.O., Hong Y., Etlioglu H.E., Cho Y.B., Pomella V., Van den Bosch B., Vanhecke J., Verbandt S., Hong H., Min J.W. et al. Lineage-dependent gene expression programs influence the immune landscape of colorectal cancer. Nat. Genet. 2020; 52:594–603. 32451460 10.1038/s41588-020-0636-z

[82] Li H., Courtois E.T., Sengupta D., Tan Y., Chen K.H., Goh J.J.L., Kong S.L., Chua C., Hon L.K., Tan W.S. et al. Reference component analysis of single-cell transcriptomes elucidates cellular heterogeneity in human colorectal tumors. Nat. Genet. 2017; 49:708–718. 28319088 10.1038/ng.3818

[83] Wu W., Zhang J., Cao X., Cai Z., Zhao F. Exploring the cellular landscape of circular RNAs using full-length single-cell RNA sequencing. Nat. Commun. 2022; 13:3242. 35688820 10.1038/s41467-022-30963-8 PMC9187688

[84] Miragaia R.J., Gomes T., Chomka A., Jardine L., Riedel A., Hegazy A.N., Whibley N., Tucci A., Chen X., Lindeman I. et al. Single-cell transcriptomics of regulatory T cells reveals trajectories of tissue adaptation. Immunity. 2019; 50:493–504. 30737144 10.1016/j.immuni.2019.01.001 PMC6382439

[85] Wu A.R., Neff N.F., Kalisky T., Dalerba P., Treutlein B., Rothenberg M.E., Mburu F.M., Mantalas G.L., Sim S., Clarke M.F. et al. Quantitative assessment of single-cell RNA-sequencing methods. Nat. Methods. 2014; 11:41–46. 24141493 10.1038/nmeth.2694 PMC4022966

[86] Cabanski C.R., Magrini V., Griffith M., Griffith O.L., McGrath S., Zhang J., Walker J., Ly A., Demeter R., Fulton R.S. et al. cDNA hybrid capture improves transcriptome analysis on low-input and archived samples. J. Mol. Diagn. 2014; 16:440–451. 24814956 10.1016/j.jmoldx.2014.03.004 PMC4078367

[87] Yang Y., Gao X., Zhang M., Yan S., Sun C., Xiao F., Huang N., Yang X., Zhao K., Zhou H. et al. Novel role of FBXW7 circular RNA in repressing glioma tumorigenesis. J. Natl Cancer Inst. 2018; 110:304–315. 28903484 10.1093/jnci/djx166 PMC6019044

[88] Lu H., Yao B., Wen X., Jia B. FBXW7 circular RNA regulates proliferation, migration and invasion of colorectal carcinoma through NEK2, mTOR, and PTEN signaling pathways *in vitro* and *in vivo*. BMC Cancer. 2019; 19:918. 31519156 10.1186/s12885-019-6028-z PMC6744671

[89] Hu Y., Zhu Y., Zhang W., Lang J., Ning L. Utility of plasma circBNC2 as a diagnostic biomarker in epithelial ovarian cancer. OncoTargets Ther. 2019; 12:9715–9723. 10.2147/OTT.S211413 PMC6859958 32009804

[90] Liu T., Yuan L., Zou X. Circular RNA circ-BNC2 (hsa_circ_0008732) inhibits the progression of ovarian cancer through microRNA-223-3p/FBXW7 axis. J. Ovarian Res. 2022; 15:95. 35965327 10.1186/s13048-022-01025-w PMC9377053

[91] Yu L., Zhu H., Wang Z., Huang J., Zhu Y., Fan G., Wang Y., Chen X., Zhou G. Circular RNA circFIRRE drives osteosarcoma progression and metastasis through tumorigenic–angiogenic coupling. Mol. Cancer. 2022; 21:167. 35986280 10.1186/s12943-022-01624-7 PMC9389772

[92] Cao G., Zhang C., Tian X., Jing G., Zhou X., Yan T. circCEP128 knockdown suppresses bladder cancer progression via regulating microRNA-515-5p/SDC1 axis. Cancer Manag. Res. 2021; 13:2885–2896. 33833571 10.2147/CMAR.S288229 PMC8020055

[93] Zhong L., Wang Y., Cheng Y., Wang W., Lu B., Zhu L., Ma Y. Circular RNA circC3P1 suppresses hepatocellular carcinoma growth and metastasis through miR-4641/PCK1 pathway. Biochem. Biophys. Res. Commun. 2018; 499:1044–1049. 29608893 10.1016/j.bbrc.2018.03.221

[94] Uhlén M., Fagerberg L., Hallström B.M., Lindskog C., Oksvold P., Mardinoglu A., Sivertsson Å., Kampf C., Sjöstedt E., Asplund A. et al. Tissue-based map of the human proteome. Science. 2015; 347:1260419. 25613900 10.1126/science.1260419

