# Supplementary material for: Characterization of cell-type specific circular RNAs associated with colorectal cancer metastasis
Source: NAR Cancer. 2023 May 19;5(2):zcad021. doi: 10.1093/narcan/zcad021 (PMC10198730; doi:10.1093/narcan/zcad021)
Supplement: zcad021_Supplemental_Files [file zcad021_supplemental_files.zip › 20230504 Supplementary Figures and Caption.pdf]

Supplementary Figure S1

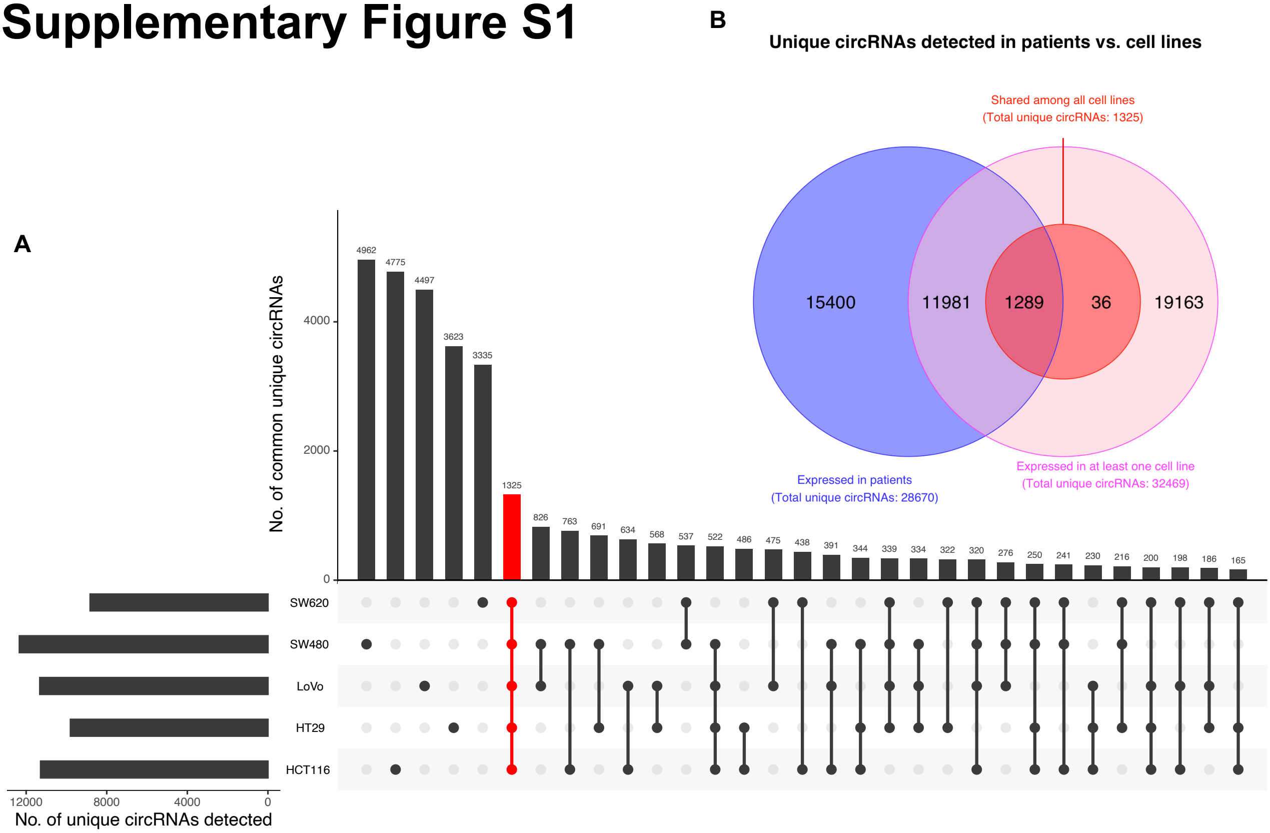

Supplementary Figure S1. Summary of circRNAs detected in WUSTL cell lines. (A) Upset plot showing unique circRNAs expressed in each cell line sample as well as those shared by samples. Each horizontal column represents the number of unique circRNAs detected in each sample. Each vertical column represents the number of shared unique circRNAs in each sample combination. Red dots denote 1 325 circRNAs that are expressed in all cell lines as the highest sample combination following circRNAs that are expressed in single samples. **(B)** Venn diagram showing the overlap between unique circRNAs detected in the patient cohort and cell line cohort. Red circle represents the 1 325 circRNAs that are shared among all cell line samples, with 1 269 of them overlapping with circRNAs in the patient cohort.

Supplementary Figure S2

A CIBERSORT proportions (Lee 2020)

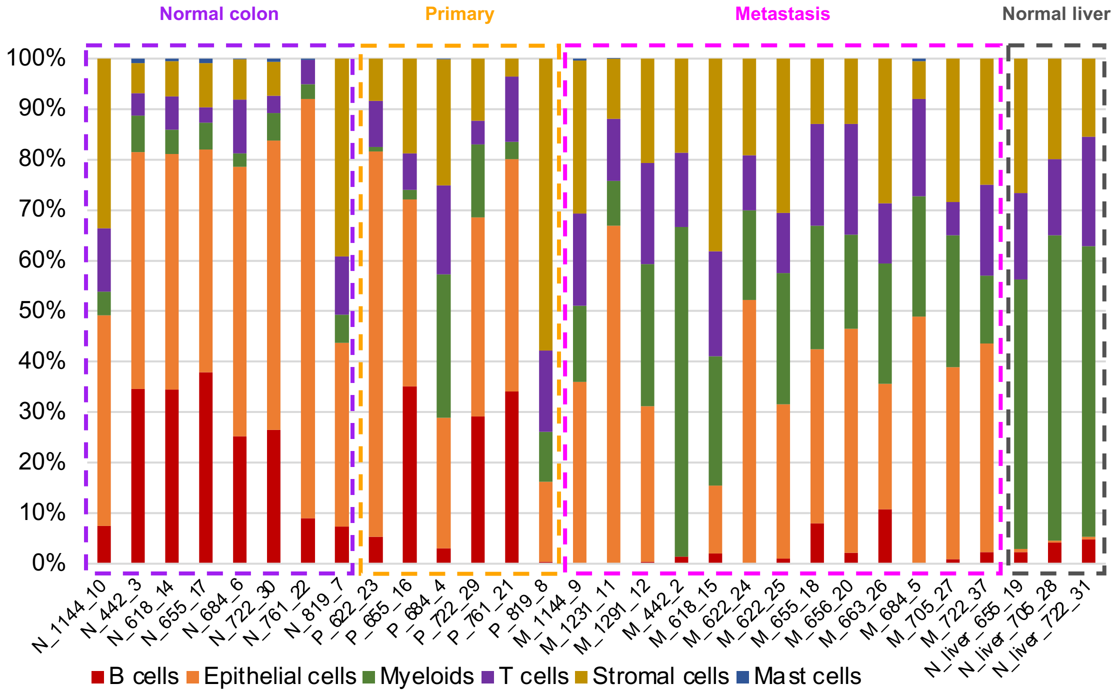

B CIBERSORT proportions (Li 2017)

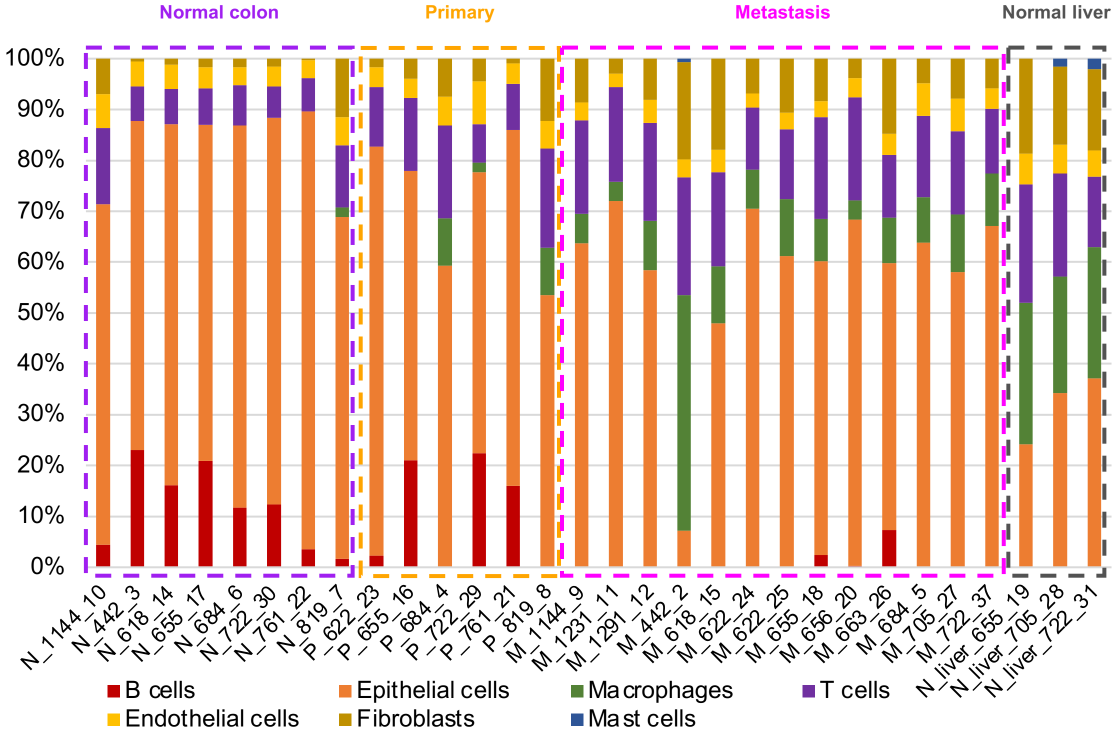

Supplement Figure S2. CIBERSORT deconvolution result from (A) the Lee 2020 cohort and (B) the Li 2017 cohort. Each column represents one patient sample. Each color in stacked columns represents one cell type, with corresponding legend below. Dashed boxes demarcate different tissue types.

Supplementary Figure S3

A

Sample	CIBERSORT Estimated Cell-Type Composition			
	Sample 1	Sample 2	……	Sample k
Cell Type 1	p_{11}	p_{12}	……	p_{1k}
……	……	……	……	……
Cell Type j	p_{j1}	p_{j2}	……	p_{jk}

Linear Gene	Bulk Expression			
	Sample 1	Sample 2	……	Sample k
Gene 1	l_{11}	l_{12}	……	l_{1k}
……	……	……	……	……
Gene i	l_{i1}	l_{i2}	……	l_{ik}

CircRNA	Bulk Expression			
	Sample 1	Sample 2	……	Sample k
CircRNA 1	n_{11}	n_{12}	……	n_{1k}
……	……	……	……	……
CircRNA i	n_{i1}	n_{i2}	……	n_{ik}

Lawson-Hanson algorithm for non-negative least squares (NNLS) model:

For the ith linear gene, the TPM (i.e. the gene expression) is modeled as:

$$l_i \approx B_{i1}p_1 + B_{i2}p_2 + \cdots + B_{ij}p_j \quad \text{where } B_{i1}, B_{i2}, \ldots, B_{ij} \geq 0$$

For the ith circRNA, the number of normalized backspliced reads (i.e. the circRNA expression) is modeled as:

$$n_i \approx C_{i1}p_1 + C_{i2}p_2 + \cdots + C_{ij}p_j \quad \text{where } C_{i1}, C_{i2}, \ldots, C_{ij} \geq 0$$

Symbol	Legend
l	Bulk linear gene expression
L	Modeled linear gene expression
n	Bulk circRNA expression
N	Modeled circRNA expression
p	CIBERSORT predicted cell-type proportion
B	NNLS model predicted cell-type specific linear gene expression coefficient
C	NNLS model predicted cell-type specific circRNA expression coefficient
i	Index for circRNA/gene
j	Index for cell type
k	Index for sample

B NNLS model benchmarking for linear genes

- Linear regression between **genes**
- Pearson correlation between **samples**

$$\begin{bmatrix} B_{11} & \cdots & B_{1j} \\ \vdots & \ddots & \vdots \\ B_{i1} & \cdots & B_{ij} \end{bmatrix} \cdot \begin{bmatrix} p_{11} & \cdots & p_{1k} \\ \vdots & \ddots & \vdots \\ p_{j1} & \cdots & p_{jk} \end{bmatrix} = \begin{bmatrix} L_{11} & \cdots & L_{1k} \\ \vdots & \ddots & \vdots \\ L_{i1} & \cdots & L_{ik} \end{bmatrix} \Longrightarrow \begin{bmatrix} l_{11} & \cdots & l_{1k} \\ \vdots & \ddots & \vdots \\ l_{i1} & \cdots & l_{ik} \end{bmatrix} \sim \begin{bmatrix} L_{11} & \cdots & L_{1k} \\ \vdots & \ddots & \vdots \\ L_{i1} & \cdots & L_{ik} \end{bmatrix}$$

NNLS coefficients
i genes, j cell types

CIBERSORT proportions
j cell types, k samples

Modeled "bulk" gene expression
i genes, k samples

Real bulk gene expression
i genes, k samples

Modeled "bulk" gene expression
i genes, k samples

C NNLS model benchmarking for circRNAs

- Linear regression between **circRNAs**
- Pearson correlation between **samples**

$$\begin{bmatrix} C_{11} & \cdots & C_{1j} \\ \vdots & \ddots & \vdots \\ C_{i1} & \cdots & C_{ij} \end{bmatrix} \cdot \begin{bmatrix} p_{11} & \cdots & p_{1k} \\ \vdots & \ddots & \vdots \\ p_{j1} & \cdots & p_{jk} \end{bmatrix} = \begin{bmatrix} N_{11} & \cdots & N_{1k} \\ \vdots & \ddots & \vdots \\ N_{i1} & \cdots & N_{ik} \end{bmatrix} \Longrightarrow \begin{bmatrix} n_{11} & \cdots & n_{1k} \\ \vdots & \ddots & \vdots \\ n_{i1} & \cdots & n_{ik} \end{bmatrix} \sim \begin{bmatrix} N_{11} & \cdots & N_{1k} \\ \vdots & \ddots & \vdots \\ N_{i1} & \cdots & N_{ik} \end{bmatrix}$$

NNLS coefficients
i circRNAs, j cell types

CIBERSORT proportions
j cell types, k samples

Modeled "bulk" circRNA expression
i circRNAs, k samples

Real bulk circRNA expression
i circRNAs, k samples

Modeled "bulk" circRNA expression
i circRNAs, k samples

Supplementary Figure S3. Benchmark of cell-type specific expression estimates from NNLS models. (A) Details of NNLS model construction using all samples. Every sample was analyzed using the method described in Figure 5A. For each sample, CIBERSORT estimated cell-type composition was tabulated. Likewise, the bulk expression profiles of linear genes and circRNAs were tabulated for all samples. The Lawson-Hanson algorithm for NNLS model was then applied for each sample to produce the estimated coefficients of cell-type specific expression for each circRNA as well as each linear gene. **(B)** For linear genes, modeled "bulk" expression (matrix *L*) of every gene in every sample is calculated through matrix multiplication of NNLS estimated coefficients (matrix *B*) and CIBERSORT estimated cell-type proportions (matrix *p*). Modeled "bulk" expression (matrix *L*) is then compared with the ground truth bulk expression (matrix *l*). A dummy linear regression is constructed between each gene to calculate p-value and R^2. Pearson correlation coefficients are calculated for each sample. **(C)** For circRNAs, modeled "bulk" expression (matrix *N*) of every circRNA in every sample is calculated through matrix multiplication of NNLS estimated coefficients (matrix *C*) and CIBERSORT estimated cell-type proportions (matrix *p*). Modeled "bulk" expression (matrix *N*) is then compared with the ground truth bulk expression (matrix *n*). A dummy linear regression is constructed between each circRNA to calculate P-value and R^2. Pearson correlation coefficients are calculated for each sample.

Supplementary Figure S4

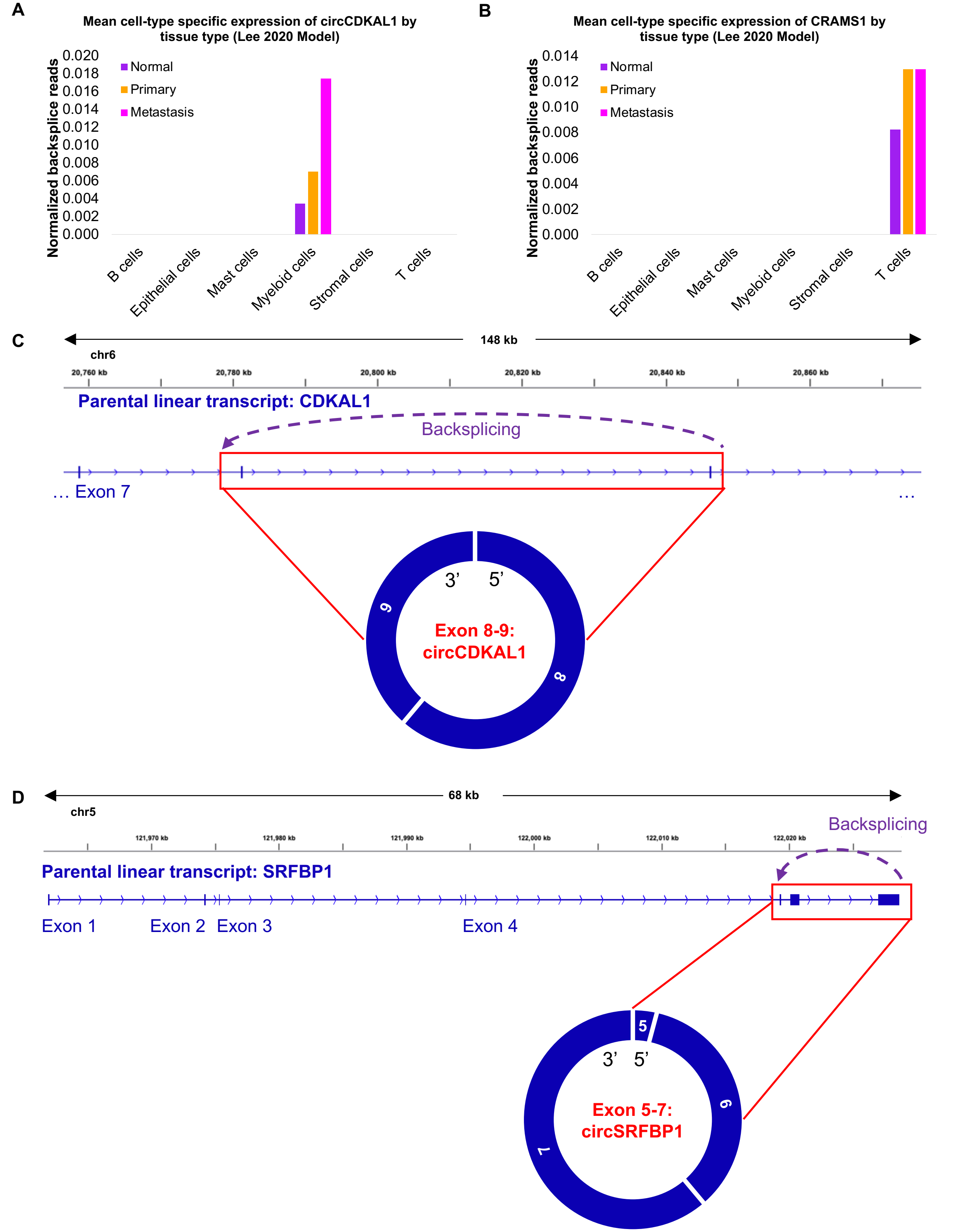

Supplementary Figure S4. Cell-type specific expression and schematic of highlight circRNAs. **(A)** Modeled cell-type specific expression of circCDKAL1 as predicted by the Lee 2020 Model. Each column represents the mean normalized expression of circCDKAL1 in each tissue type, showing upregulation from normal to metastasis that is specific in myeloid cells. **(B)** Modeled cell-type specific expression of CRAMS1 as predicted by the Lee 2020 Model. Each column represents the mean normalized expression of CRAMS1 in each tissue type, showing upregulation from normal to primary that is specific in T cells. **(C)** Schematic representation circCDKAL1. Parts of the parental gene CDKAL1 is shown below in blue, with its exon 8-9 constituting circCDKAL1. **(D)** Schematic representation circSRFBP1. The full-length parental gene SRFBP1 is shown below in blue, with its exon 5-7 constituting circSRFBP1.
